# Novel perspectives of target-binding by the evolutionarily conserved PP4 phosphatase

**DOI:** 10.1098/rsob.200343

**Published:** 2020-12-23

**Authors:** Zoltan Karman, Zsuzsanna Rethi-Nagy, Edit Abraham, Lilla Fabri-Ordogh, Akos Csonka, Peter Vilmos, Janusz Debski, Michal Dadlez, David M. Glover, Zoltan Lipinszki

**Affiliations:** 1 Biological Research Centre, Institute of Biochemistry, MTA Lendület Laboratory of Cell Cycle Regulation, Szeged, H‐6726, Hungary; 2 Doctoral School of Biology, Faculty of Science and Informatics, University of Szeged, Szeged, H‐6725, Hungary; 3 Department of Traumatology, University of Szeged, Szeged, H‐6725, Hungary; 4 Biological Research Centre, Institute of Genetics, Szeged, H‐6726, Hungary; 5 Laboratory of Mass Spectrometry, Institute of Biochemistry and Biophysics, Polish Academy of Sciences, 02-106 Warsaw, Poland; 6 Department of Genetics, University of Cambridge, Cambridge CB2 3EH, UK; 7 California Institute of Technology, Pasadena, CA 91125, USA

**Keywords:** PP4, EVH1, Smk-1, SLiM, binding motif, *Drosophila*

## Abstract

Protein phosphatase 4 (PP4) is an evolutionarily conserved and essential Ser/Thr phosphatase that regulates cell division, development and DNA repair in eukaryotes. The major form of PP4, present from yeast to human, is the PP4c-R2-R3 heterotrimeric complex. The R3 subunit is responsible for substrate-recognition via its EVH1 domain. In typical EVH1 domains, conserved phenylalanine, tyrosine and tryptophan residues form the specific recognition site for their target's proline-rich sequences. Here, we identify novel binding partners of the EVH1 domain of the *Drosophila* R3 subunit, Falafel, and demonstrate that instead of binding to proline-rich sequences this EVH1 variant specifically recognizes atypical ligands, namely the FxxP and MxPP short linear consensus motifs. This interaction is dependent on an exclusively conserved leucine that replaces the phenylalanine invariant of all canonical EVH1 domains. We propose that the EVH1 domain of PP4 represents a new class of the EVH1 family that can accommodate low proline content sequences, such as the FxxP motif. Finally, our data implicate the conserved Smk-1 domain of Falafel in target-binding. These findings greatly enhance our understanding of the substrate-recognition mechanisms and function of PP4.

## 1. Introduction

Protein phosphorylation serves as a molecular switch to regulate the activity, subcellular localization, interacting partners, structure and half-life of proteins. It is mediated by protein kinases and reversed by protein phosphatases. Protein phosphatases can be classified into four major groups, of which the Ser/Thr phosphoprotein phosphatases (PPPs) (including PP1, PP2A, PP2B, PP4, PP5, PP6 and PP7) confer more than two thirds of phosphatase activity in the eukaryotic cell [1]. To counteract the function of the large number of kinases (approx. 400) [2], most of the catalytic subunits (approx. 40) of the PPP family associate with regulatory proteins and form different holoenzymes with distinct functions [3,4]. To better understand phosphoregulation-mediated cell signalling, it is important to define the modular composition of the various PPP holoenzymes, and to identify the mode of substrate-binding for their different catalytic (c) and regulatory (R) subunits.

**Figure 1.**
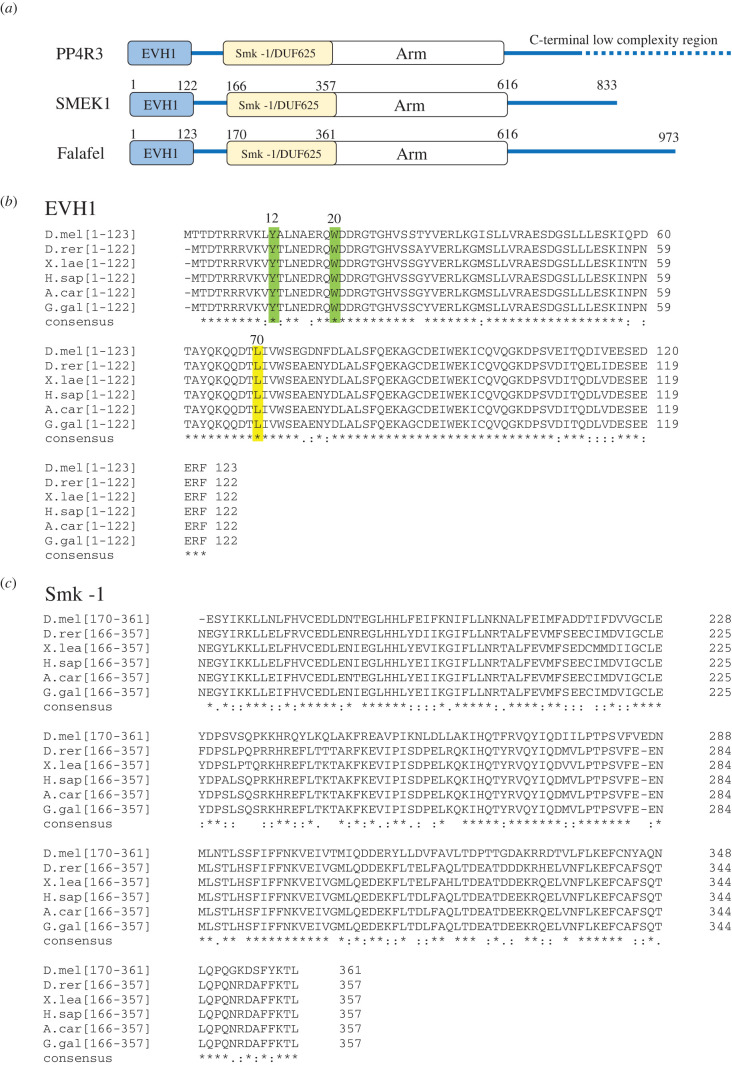
Schematic of PP4R3 subunits and sequence alignments of the conserved EVH1 and Smk-1 domains. (*a*) PP4R3 orthologues share similar domain architecture. The conserved EVH1 domain occupies the N-terminal end, which is followed by the conserved Smk-1/DUF625 domain and a variable number of Armadillo/HEAT (Arm) repeats. The C-termini of PP4R3 orthologues bear low complexity regions. SMEK1 is the human, while Falafel is the fruit fly counterpart of PP4R3. (*b*) Multiple sequence alignments of the EVH1 domains of PP4R3 orthologues in Metazoa. Tyr12 (Y) and Trp20 (W) highlighted in green as well as Leu70 (L) highlighted in yellow (*Drosophila* numbering) are conserved residues that are essential for target-binding. (*c*) Multiple sequence alignments of the Smk-1 domains of PP4R3 orthologues in Metazoa. D.mel (*Drosophila melanogaster* Falafel, Uniprot: Q9VFS5); D.rer (*Danio rerio* SMEK1, Uniprot: Q5SP90), X.lae (*Xenopus laevis* SMEK1, Uniprot: Q6INN7), H.sap (*Homo sapiens* SMEK1, Uniprot: Q6IN85), A.car (*Anolis carolinensis* PPP4R3A, Uniprot: G1KJ53) and G.gal (*Gallus gallus* PP4R3, Uniprot: F1NPW9). Consensus sequence symbols: * (asterisk) indicates conserved residues; : (colon) indicates strongly similar residues; . (period) indicates weakly similar residues.

Protein phosphatase 4 is an essential PP2A-like phosphoprotein phosphatase (reviewed in [5]), which plays an important role in the regulation of the cell cycle [6–12], DNA repair [13–18], cell death [19,20] and differentiation [21,22]. PP4 often functions as a heterotrimeric holoenzyme consisting of an evolutionarily conserved catalytic subunit (PP4c) that associates with a scaffolding subunit (PP4R2, R2) and a regulatory-3 subunit (PP4R3, R3) [5,23–25]. Although in higher eukaryotes PP4c can form additional, mutually exclusive complexes, the major form of the enzyme, which is present from yeast to human, is the PP4c-R2-R3 heterotrimeric complex (hereafter PP4). PP4R3 orthologues (Psy2 in yeast [26,27], Falafel (Flfl) in *Drosophila* [8,28] and PP4R3*α*/SMEK1 and PP4R3*β*/SMEK2 in mammals [24]) are highly conserved, share similar domain architecture and are responsible for substrate recognition and subcellular localization of the holoenzyme [5,16,22,29–32]. PP4R3 orthologues contain two highly conserved domains occupying the N-terminal region of the protein (figure 1); an EVH1 domain, which belongs to the pleckstrin homology (PH) superfamily-like domains [33], and an Smk-1 domain of unknown function (DUF625). The N-terminal domains are followed by Armadillo (ARM/HEAT) repeats in the middle, and a low complexity tail region of various lengths in the C-terminal parts of the orthologues (figure 1*a*).

We and others have shown that the EVH1 domain of the PP4R3 orthologues confers substrate specificity by directly interacting with the target proteins. For example, the EVH1 domain of the yeast PP4R3^Psy2^ interacts with the **MPPP** short linear motif (SLiM) of the glucose signal transducer protein Mth1 [30], suggesting that PP4R3^Psy2^ binds to proline-rich sequences (PRS), similar to canonical EVH1 proteins [33,34]. However, we reported previously that in *Drosophila* the EVH1 domain of PP4R3^Flfl^ specifically binds to the low-proline 19-mer Falafel-Interacting Motif (FIM) of the key centromeric protein, CENP-C [8]. This work revealed for the first time that the EVH1 domain of Falafel can also recognize ligands with low proline content, such as the **FKKP** (Phe-Lys-Lys-Pro) sequence within the FIM [8]. Recently, it has been reported that the EVH1 domain of the human PP4R3*α*^SMEK1^ recognizes both poly-proline-like (MxPP) and low-proline (FxxP) consensus binding motifs [32]. Here, we report on the identification of novel interacting partners of the *Drosophila* PP4R3^Flfl^ and demonstrate that Falafel^EVH1^ directly binds to the targets' FxxP and MxPP SLiMs, similar to its human counterpart. This further supports the idea that PP4 uses the same SLiMs for substrate recognition across species. This interaction is dependent on the Leu70 (Leu69 in humans) residue of the EVH1 domain, which is exclusively conserved in all PP4R3 orthologues. Finally, we resolve the previously unknown function of the Smk-1 domain of PP4R3 and demonstrate that it is physically involved in target recognition in flies.

## 2. Results

### 2.1. Identification of novel targets of PP4R3^Flfl^

PP4 is involved in the regulation of cell division, which includes centrosome function, microtubule organization, spindle assembly checkpoint activity and kinetochore integrity [5]. However, the molecular mechanisms involved in its regulatory activities remain poorly defined. Therefore, we aimed to use affinity purification coupled to mass spectrometry (AP-MS) to identify novel targets of the *Drosophila* PP4R3^Flfl^ (hereafter Falafel or Flfl) involved in cell cycle control. Our repeated attempts to identify new binding partners of transgenic Falafel purified from D.Mel-2 cultured cells (representing interphase cells) or *Drosophila* syncytial embryos, which characteristically undergo rapid and synchronous nuclear divisions (representing mitotically active tissue), were not successful, with only few previously identified interactors detected. As transgenic Falafel forms a functional complex with R2 and PP4c [8], we have hypothesized that the interaction between the target protein and PP4c-R2-Falafel could be transient, with the substrate being released upon dephosphorylation.

To overcome this possible limitation, we engineered truncated forms of Falafel: Flfl^1-168aa^ (containing the EVH1 domain, hereafter EVH1) and Flfl^169-361aa^ (containing the Smk-1 domain, hereafter Smk-1) each fused to glutathione S-transferase (GST). Then we performed GST pull-down from wild-type syncytial embryos followed by mass spectrometric (MS) analysis of the samples. This strategy resulted in the identification of several putative interacting partners of Falafel (electronic supplementary material, table S1). These included expected known PP4 substrates such as barrier-to-autointegration factor (BAF) [10,12], CENP-C [8], Miranda [22] and gamma-tubulin [35] as well as previously unrecognized proteins, many of which are predicted to be phosphoregulated and involved in cell division/cell cycle control, development or DNA repair. This included all four members of the chromosomal passenger complex (CPC), a key regulator of mitosis (reviewed in [36,37]). Consistent with previous work most interactors were bound to the EVH1 domain.

Surprisingly, the Smk-1 domain also pulled-down several putative partners of Falafel (electronic supplementary material, table S1), including the gamma-tubulin ring complex proteins known to be essential for spindle microtubule nucleation at the centrosomes (reviewed in [38]). In addition, we identified all three members of the conserved Rod-Zwilch-Zw10 (RZZ) complex. The RZZ is regulated by phosphorylation, and its activity stabilizes the kinetochore association of Mad1 and Mad2 spindle assembly checkpoint factors, thus ensuring accurate chromosome segregation. Smk-1 also co-purified with the heavy chain of the motor protein dynein, a previously identified RZZ interactor and a factor involved in the transport of Mad2 from kinetochores [39,40]. These findings suggest that PP4 directly regulates the spindle assembly checkpoint activity for faithful chromosome segregation and that the conserved Smk-1 domain may also be responsible for target recognition.

### 2.2. Identification of novel PP4R3-EVH1 domain interacting proteins

**Figure 2.**
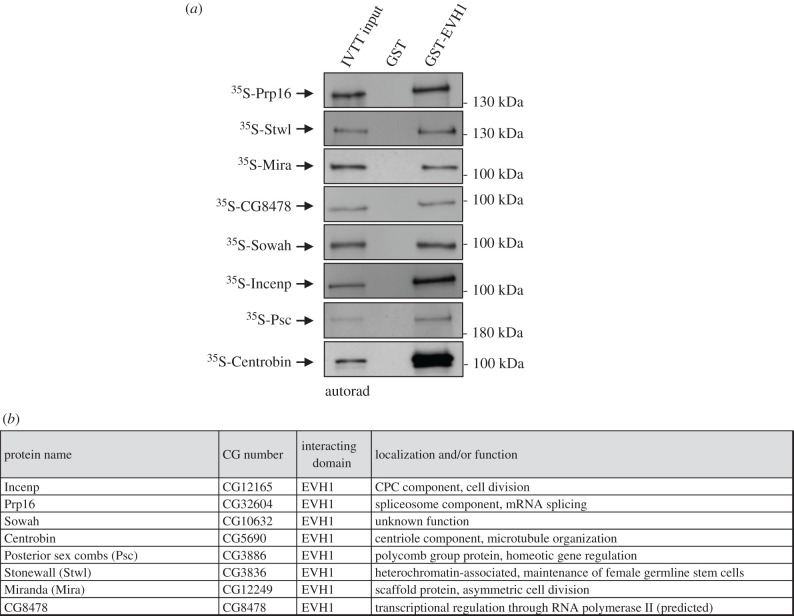
Proteins physically interacting with the EVH1 domain of Falafel. (*a*) Autoradiographs of *in vitro* binding of GST-tagged EVH1 with IVTT-produced ^35^S-methionine-labelled Prp16, Stwl, Mira, CG8478, Sowah, Incenp, Psc and Centrobin prey proteins. GST served as a negative control. Coomassie Brilliant Blue-stained gels are shown in electronic supplementary material, figure S1B. (*b*) Table summarizing the name, CG number (FlyBase annotation IDs), function and/or localization of the EVH1-interacting proteins.

We next sought to confirm the physical interactions between the EVH1 domain of Falafel and the AP-MS-identified putative target proteins (electronic supplementary material, table S1). We selected several proteins from the list and performed *in vitro* protein–protein interaction (PPI) experiments. Recombinant GST or GST-EVH1 were immobilized on glutathione sepharose 4B beads (electronic supplementary material, figure S1A) and mixed with ^35^S-methionine-labelled prey proteins generated by coupled *in vitro* transcription and translation reactions (IVTT). Following SDS-PAGE and autoradiography, 8 proteins were validated as direct interactors of EVH1 (figure 2; electronic supplementary material, figure S1B).

From the other tested proteins, a few did not express in IVTT or showed no interaction with the EVH1 domain in the PPI experiments (electronic supplementary material, figure S2A). The source of the discrepancy remains to be determined. While we cannot rule out contamination, at least some of the proteins identified *in vivo* may represent indirect interactions. This can be seen for example with CPC components. While the entire CPC complex was identified by AP-MS, only the localization domain protein Incenp bound to EVH1 directly in the PPI assay. This suggests that PP4 binds to the CPC via Incenp. The heterochromatin-associated BAF, a known substrate of PP4 [10,12], was also found in our AP-MS screen as an EVH1 interactor. However, no physical interaction with EVH1 (or Smk-1, see below) was detected (electronic supplementary material, figure S2A,B). In a recent study, we have shown that a small pool of BAF is also present at the centromere (cenBAF) where it associates with CENP-C via a PP4-dependent mechanism [12]. Given the precedent of CENP-C's robust interaction with PP4-R2-Falafel (electronic supplementary material, table S1 and figure 4*b*) [8], we propose that CENP-C serves as a platform for PP4, allowing it to dephosphorylate proteins that target to the centromere but which it does not directly recruit, such as BAF.

### 2.3. The EVH1 domain preferentially binds to the FxxP and MxPP consensus motifs

Canonical EVH1 proteins establish interactions with their ligands by recognizing polyproline motifs [34]. Accordingly, the EVH1 domain of the yeast PP4R3^Psy2^ binds to the proline-rich **MPPP** SLiM of the Mth1 protein for glucose signalling regulation [30]. By contrast, we previously demonstrated that the EVH1 domain of PP4R3^Flfl^ could bind to the low proline content **FKKP** motif localized in the FIM region of CENP-C and that mutation of either the F (Phe) or P (Pro) residues abolished the interaction [8].

We therefore searched for motifs similar to FKKP and MPPP in the primary sequence of the newly identified direct interacting partners of EVH1. We found various numbers (from 1 to 7) of sequences similar to FKKP or MPPP (electronic supplementary material, figure S3A), suggesting that the consensus recognition sequences might be **F**xx**P** and **M**x**PP**. Most EVH1-targets contain several putative SLiMs, such as Prp16, which has 6 FxxP and 1 MxPP motif. However, CG8478 has a single MAPP (aa 504–507) motif. Our analysis of the adjacent sequences of several FxxP and MxPP motifs failed to establish any pattern that predicts which motif is recognized by the EVH1 domain. We therefore investigated the putative binding regions of 7 different EVH1-target proteins (Prp16, Psc, Incenp, Stwl, Sowah, CG8478 and Miranda) that interact with EVH1, *in vitro*. We generated a variable number of ^35^S-methionine-labelled partially overlapping sequences of the target proteins by IVTT: 6 for Prp16, 7 for Psc, 6 for Incenp, 5 for Stwl, 5 for Sowah and 5 for CG8478. We made a single recombinant protein for Miranda spanning aa 1–280, a region previously reported to bind Falafel [22] and that contains 2 FxxP motifs. We were unsuccessful at expressing Centrobin pieces by IVTT. Next, we performed *in vitro* PPI experiments using the different target fragments as prey and GST or GST-EVH1 as bait. The binding results were analysed by SDS-PAGE followed by autoradiography (electronic supplementary material, figure S4), allowing us to identify the minimally required EVH1-interacting fragments in most cases.

**Figure 3.**
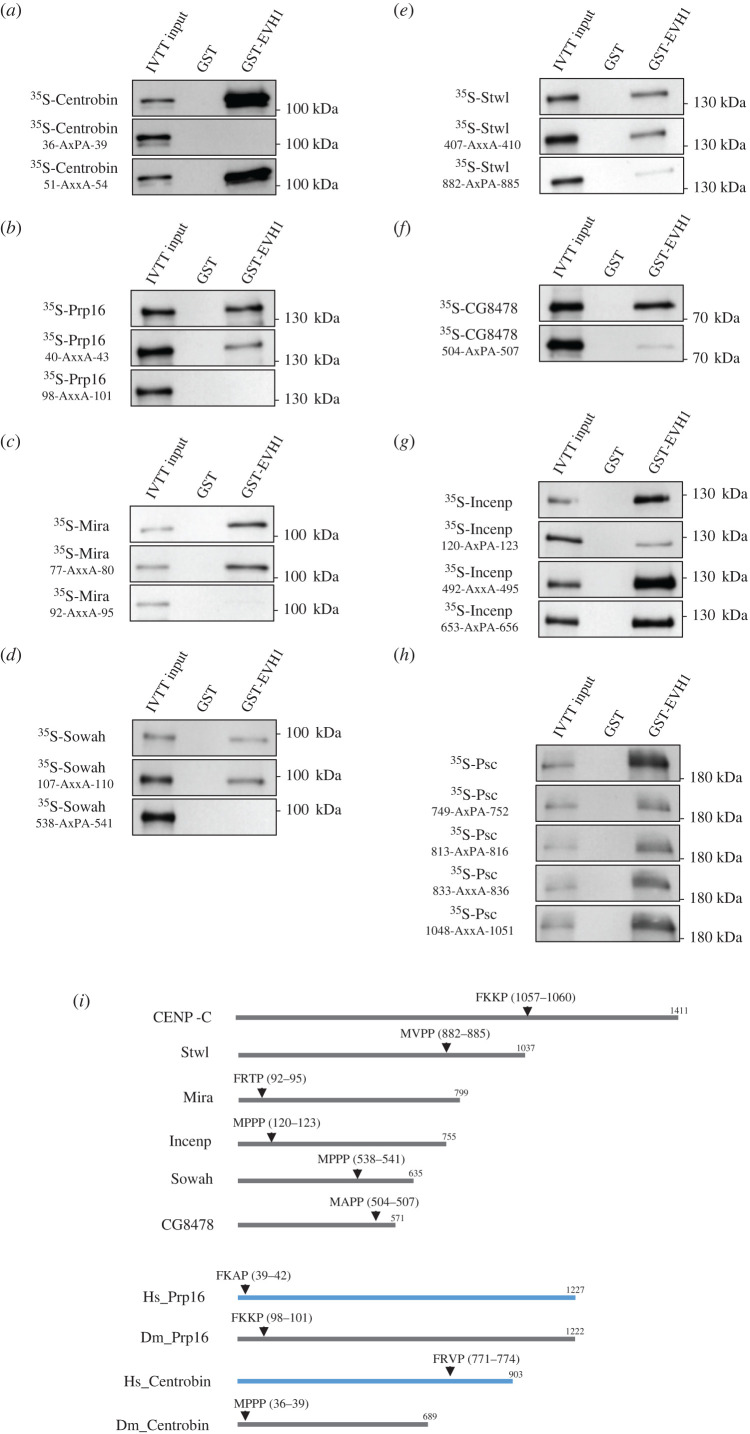
Identification of the EVH1-binding FxxP or MxPP SLiMs. Autoradiography images of *in vitro* binding of GST-EVH1 and IVTT-expressed ^35^S-labelled wild-type or mutated (FxxP to AxxA, MxPP to AxPA) full length Centrobin (*a*), Prp16 (*b*), Mira (*c*), Sowah (*d*), Stwl (*e*), CG8478 (*f*) or Incenp (*g*). (*h*) Mutation of the putative FxxP/MxxP motifs in Psc did not alter the interaction with GST-EVH1. (*i*) Schematic of the sequences and location (amino acid numbers are in parentheses) of the identified EVH1-binding SLiMs in eight different Falafel-interacting proteins. *Drosophila* (Dm, grey lines) and human (Hs, blue lines, according to [32]) Prp16 and Centrobin are shown separately. Full sequences and putative SLiMs of the *Drosophila* proteins are shown in electronic supplementary material, figure S3A.

To further determine which motifs are responsible for EVH1-binding we mutated the FxxP or MxPP motifs to AxxA (A, alanine) (for FxxP) and AxPA (for MxPP) in the full-length proteins and assayed them by PPI. We found that CG8478's **MAPP** (aa 504–507), Miranda's **FRTP** (aa 92–95), Prp16's **FKKP** (aa 98–101), Sowah's **MPPP** (aa 538–541) and Stwl's **MVPP** (aa 882–885) sequences are essential for EVH1 binding, whereas mutations of other FxxP/MxPP sequences did not alter the interaction (figure 3*b–f*). Interestingly, each of these targeting sequences lay in structurally disordered regions (electronic supplementary material, figure S3A). We further found that out of 2 putative SLiMs, Centrobin uses the **MPPP** (aa 36–39) for EVH1 binding (figure 3*a*). Examination of Incenp revealed that although it has 3 putative SLiMs, only mutation of the **MPPP** (aa 120–123) to AxPA weakened the interaction with EVH1 (figure 3*g*). We also managed to map the 2 interacting fragments of Psc that bind to EVH1 (aa 721–810 and aa 786–876; electronic supplementary material, figure S4F). However, mutagenesis of the 3 putative SLiMs within, and 1 outside of these regions failed to alter their interaction with EVH1 (figure 3*h*). This suggests that Psc binds to EVH1 in an FxxP/MxPP SLiM-independent manner. We were unable to challenge the function of the fifth motif (1201-MTPP-1204) as the protein did not express in IVTT reaction. These experiments unequivocally reveal that most of the EVH1-interacting partners we have identified bind to EVH1 via their FxxP or MxPP short linear motifs.

**Figure 4.**
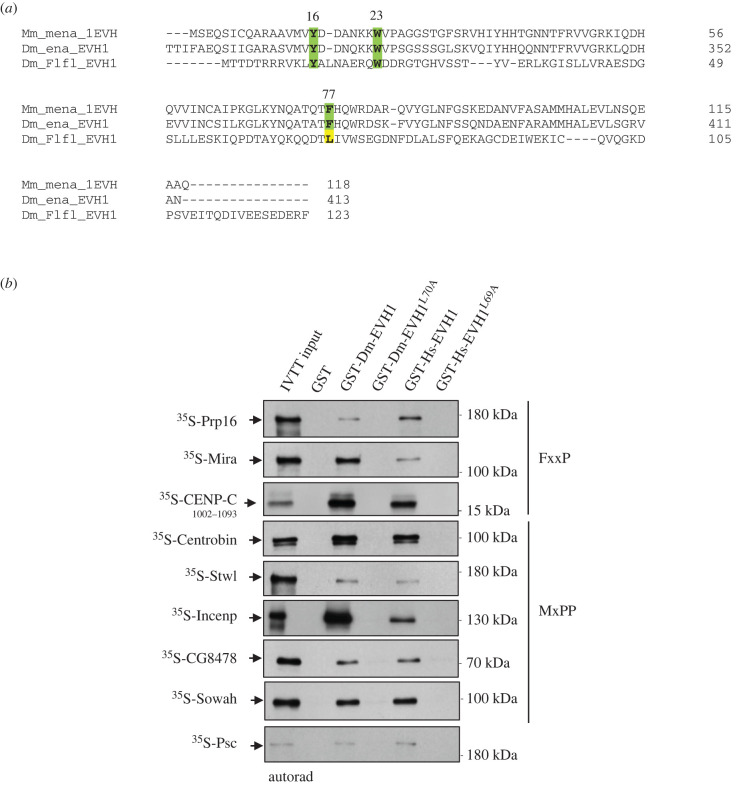
Leu70/69 is essential for target-binding in fruit flies and humans. (*a*) Multiple sequence alignment of the EVH1 domains of murine mena (Mm, Uniprot: Q03173), fruit fly ena (Dm, Uniprot: Q8T4F7) and fruit fly PP4R3/Falafel (Dm, Uniprot: Q9VFS5) proteins. While Tyr16 (Y) and Trp23 (W) (mena numbering) highlighted in green are highly conserved in all proteins, Phe77 (F) highlighted in green is replaced by Leu (L) in Falafel (highlighted in yellow). (*b*) Autoradiographs of *in vitro* binding of GST-tagged Dm-EVH1, Dm-EVH1^L70A^, Hs-EVH1 or Hs-EVH1^L69A^ with IVTT-produced ^35^S-methionine-labelled prey proteins. GST served as a negative control. Coomassie Brilliant Blue-stained gels are shown in electronic supplementary material, figure S1D.

Intriguingly, Prp16 and Centrobin are found to be targets of the EVH1 domain of *Drosophila* Falafel (figure 2*a*) and human SMEK1, as well [32]. Although Centrobin is functionally conserved in both species, there is no significant primary sequence homology between the two orthologues (electronic supplementary material, figure S5A). Similarly, besides the conserved RNA helicase domain occupying the C-terminal region of Prp16, the N-terminal unstructured halves of the fruit fly and human orthologues do not show a high level of primary sequence conservation (electronic supplementary material, figure S5B). Prp16 is recognized by the PP4R3-EVH1 domain via its N-terminally located FKKP (aa 98–101) SLiM in *Drosophila* and via its FKAP (aa 39–42) motif in humans, respectively (figure 3*b,i*; electronic supplementary material, figure S5B). Neither SLiM is conserved. While Centrobin is bound by the EVH1 domain via its N-terminally located MPPP (aa 36–39) motif in *Drosophila,* it is recognized via the C-terminally positioned FRVP (aa 741–774) SLiM sequence in humans (figure 3*a,i*; electronic supplementary material, figure S5A). However, the human EVH1 of SMEK1 is able to bind to *Drosophila* Centrobin and Prp16 proteins (figure 4*b*). These observations confirm that PP4 has conserved targets and it recognizes FxxP or MxPP SLiMs via the EVH1 domain of its R3 subunit. However, the position and composition of the SLiMs may differ even in the same orthologues.

Taken together with previous [8] findings, we propose that the EVH1 domain of the *Drosophila* PP4R3^Flfl^ subunit preferentially binds to the **FRTP** and **FKKP** as well as the **MPPP**, **MAPP** or **MVPP** short linear motifs. In humans, the EVH1 domain of the SMEK1 also preferentially binds FxxP and MxPP short motifs [32], suggesting that the substrate-binding mode of the PP4R3 orthologues' EVH1 domain is conserved throughout species.

### 2.4. A conserved leucine in EVH1 of PP4R3 orthologues is critical for SLiM-binding in fruit flies and humans

Crystallographic studies have revealed that the low proline content CENP-C^FIM^ (1048-PDESSADVV**FKKP**LAPAPR-1068) is recognized by the EVH1 domain of Falafel in a polyproline II (PPII [33]) conformation and that the essential Phe1057 (F1057) occupies a hydrophobic pocket in EVH1, whereas Pro1060 (P1060) is sandwiched between the Leu70 and the conserved Trp20 residues of Falafel [8]. Through sequence alignments of EVH1 domains we demonstrated that Phe77 (1EVH numbering of murine mena [41]), which is highly conserved and invariant in all known canonical EVH1 domains and essential for ligand-binding [33], is replaced by leucine (Leu70) in Falafel^EVH1^ (figure 4*a*). More interestingly, the Leu70 (Falafel numbering) is exclusively conserved in PP4R3 orthologues, from yeast (Leu91 in Psy2) to human (Leu69 in SMEK1 and SMEK2) (figure 1*b*; electronic supplementary material, figure S5C). This suggests that the EVH1 domain of PP4R3 orthologues deviates from the canonical EVH1 domains and uses an invariant leucine residue in binding to atypical (low proline content) ligands, such as the FxxP (or MxPP) SLiM.

To test this assumption, we substituted the Leu70 in Falafel's EVH1 to alanine (hereafter GST-Dm-EVH1^L70A^) and Leu69 in human SMEK1's EVH1 to alanine (hereafter GST-Hs-EVH1^L69A^) and tested their interactions with 9 different Falafel^EVH1^-interacting *Drosophila* proteins, *in vitro*. We found that not only was *Drosophila* EVH1 able to bind to these targets, but the human EVH1 also interacted with each of the *Drosophila* proteins (figure 4*b*). Thus, target recognition is conserved across species. In both cases the binding was dependent upon the conserved Leu70/69 residues with the alanine substitutions completely abolishing interaction with the target FxxP or MxPP SLiM-containing proteins (figure 4*b*). Strikingly, *Drosophila* Prp16 and Centrobin were bound to the human SMEK1's EVH1 despite their human counterparts having different SLiM recognition sequences (figure 3*i*; electronic supplementary material, figure S5A-B). Psc was also able to bind to Dm-EVH1 and Hs-EVH1, which was dependent on Leu70/Leu69 residues, respectively (figure 4*b*).

### 2.5. The Smk-1 domain of Falafel is involved in target-binding

**Figure 5.**
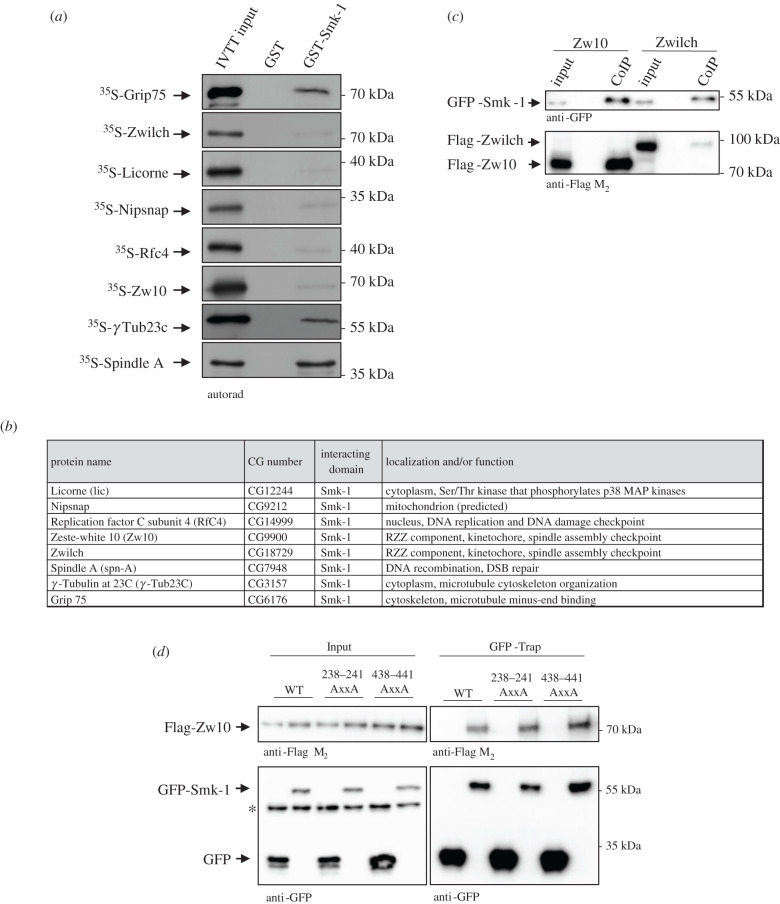
The Smk-1 domain of Falafel interacts with a new set of proteins in an FxxP- and EVH1-independent manner. (*a*) Autoradiographs of *in vitro* binding of GST-tagged Smk-1 with IVTT-produced ^35^S-methionine-labelled Grip75, Zwilch, Licorne, Nipsnap, Rfc4, Zw10, γ‐Tub23C and Spindle A prey proteins. GST served as a negative control. Coomassie Brilliant Blue-stained gels are shown in electronic supplementary material, figure S1C. (*b*) Table summarizing the name, CG number (FlyBase annotation IDs), function and/or localization of the Smk-1-interacting proteins. (*c*) GFP-Smk-1 transiently co-expressed with Flag-Zw10 or Flag-Zwilch in D.Mel-2 cultured cells was GFP-Trapped and analysed by SDS-PAGE followed by immunoblotting using anti-GFP and anti-FlagM_2_ antibodies. Flag-Zw10 strongly binds to GFP-Smk-1 *in vivo*. (*d*) GFP-Smk-1 transiently co-expressed with Flag-Zw10, Flag-Zw10(^238^AxxA^241^) or Flag-Zw10(^438^AxxA^441^) in D.Mel-2 cultured cells was GFP-Trapped and analysed by SDS-PAGE followed by immunoblotting using anti-GFP and anti-FlagM_2_ antibodies. Mutation of the two FxxP motifs did not alter the interaction between Smk-1 and Zw10. Asterisk (*) indicates a non-specific band recognized by the antibody.

The Smk-1/DUF625 domain was first identified in the SMK-1 protein, a component of the IIs longevity pathway in *C. elegans* [31]. SMK-1 is the R3 subunit of PP4, which together with Daf-16 contributes to innate immunity in adult roundworms [42]. The SMK-1 domain is present in PP4R3 orthologues from yeast to humans and its primary sequence is highly conserved in Metazoa (figure 1*c*). However, its function and structure are currently unknown. Our AP-MS experiment identified several putative targets of Falafel's Smk-1 domain including components of the RZZ complex, additional spindle assembly checkpoint (SAC) proteins and DNA repair determinants. We selected several candidates and tested whether they directly interact with the Smk-1 domain of Falafel using IVTT and GST-Smk-1 immobilized onto beads. This confirmed that 8 of the proteins physically interact with Smk-1 (figure 5*a,b*). Of the remaining candidates, one was not expressed in IVTT (electronic supplementary material, table S1) and a few failed to bind *in vitro* (electronic supplementary material, figure S2B). Although among the RZZ components both Zw10 and Zwilch showed weak but specific interactions with GST-Smk-1 *in vitro* (figure 5*a*), in D.Mel-2 cultured cells Flag-Zw10 showed stronger interaction with GFP-Smk-1 (figure 5*c,d*), suggesting that Zw10 may link Falafel/PP4 to the RZZ complex. Even though we used equal amounts of GST-EVH1 or Smk-1 in the *in vitro* PPI experiments (electronic supplementary material, figure S1A-C), we noticed that Smk-1 shows a weaker binding to its targets (figure 5*a*) compared to the binding of EVH1 to its own targets (figure 2*a*). However, we consider most of the Smk-1 interactions specific, because neither GST nor GST-EVH1 could interact with the binding partners of Smk-1 (electronic supplementary material, figure S2A).

Although neither the 3D structure nor mode of ligand-binding of the Smk-1 domain has been revealed so far, it is not reasonable to suppose that it recognizes the same SLiMs as the EVH1 domain does. Indeed, among the 8 identified direct interactors of Smk-1, 7 proteins do not contain FxxP or MxPP motifs at all (electronic supplementary material, figure S3B). Zw10 is the only one to contain FxxP sequences (aa 238–241 and 438–441), whose mutation to AxxA did not abolish the interaction between GFP-Smk-1 and Flag-Zw10 in D.Mel-2 cultured cells (figure 5*d*). This suggests that Smk-1 uses a completely different, but still unknown mechanism for target recognition. Our attempts to identify consensus binding-motifs within the full-length proteins or narrow-down Smk-1-interacting regions of the target proteins (electronic supplementary material, figure S6) have failed so far.

## 3. Discussion

According to Paul Ehrlich's key-lock principle, agents only work when they are bound (*Corpora non agunt nisi fixata*) [43]. This rule also applies to phosphatases, which must recognize and physically bind to their targets to catalyse their dephosphorylation. It is well established that PP1, PP2A and PP2B/Calcineurin Ser/Thr PPPs use their catalytic or regulatory domains to recognize short linear motifs (SLiMs) in their target substrate proteins. The catalytic subunit of PP1 directly interacts with the RVxF-type docking motif [44,45], which might be supported by the N-terminally localised SILK or MyPhoNe sequences [46]. The catalytic subunit of PP2B/Calcineurin directly binds to its SLiMs (LxVP and PxIxIT), too, independently of the regulatory domain [47,48]. However, the SLiM preference of PP2A is determined by its various regulatory subunits: B56 recognizes the LxxIxEx/LSPIxE motif [49,50], while B55 binds its substrates differently [51]. By contrast, comparatively little is reported about the mechanisms employed by other PPP family members (PP4–7) to target their substrates.

In higher eukaryotes, the PP4c catalytic subunit of PP4 can form multiple, mutually exclusive complexes with differing regulatory subunits, including the heterodimeric PP4c-R1, PP4c-R4 as well as the heterotrimeric PP4c-R2-R3, which is the major form and is present from yeast to humans (reviewed in [5]). It is believed that the regulatory R3 subunit directly interacts with PP4 substrates through its N-terminal EVH1 domain, but the target SLiM has not been identified. Since canonical EVH1 domains bind to proline-rich sequences, it was reasonable to suppose that the R3 orthologues bind to high proline content SLiMs, too. The discovery that yeast R3^Psy2^ interacts with the MPPP sequence of the glucose signal transducer protein Mth1 [30] had partially supported this idea. However the MPPP SLiM was slightly different from typical EVH1 ligands [33,34]. We previously reported that the EVH1 domain of the R3 subunit (Falafel) of the *Drosophila* PP4 can specifically bind to low-proline sequences, as well. For the first time, we demonstrated that the EVH1 preferentially bound to the FKKP-containing FIM motif of CENP-C, in which the F and P were essential for the interaction [8]. Here, we describe the discovery of novel physical interactors of the EVH1 domain of Falafel. We have identified several putative SLiMs similar to the FKKP and MPPP sequences and demonstrate that the consensus binding motifs recognized by PP4's EVH1 domain are FxxP and MxPP. A recent finding was also made using the human PP4R3^SMEK1^ [32], revealing that the binding mechanism of PP4 has been highly conserved throughout evolution. This is further supported by our observation that the human SMEK1-EVH1 is able to bind to all *Drosophila* proteins that are direct interactors of Falafel-EVH1, including Prp16 and Centrobin. Remarkably, *Drosophila* and human Centrobin are only functional homologues, and the PP4-bound FRVP SLiM present in the human protein [32] is different from the MPPP sequence we identified in its fruit fly counterpart. We have identified a similar interchangeability for Prp16. From these and other complementary data, we propose that not only the binding mechanism of PP4 but also its substrate set and accompanying regulatory roles are conserved and that this is irrespective of sequence similarity or target protein SLiMs. Indeed, FxxP and MxPP sequences are quite frequent even in the simple proteomes, raising the likelihood of PP4-mediated regulation. Given the short sequence length it is unclear how FxxP or MxPP motifs are selected by PP4. It is unlikely to require unique topological cues as these sequences commonly reside in disordered regions of the protein. Future work will be required to define this mechanism.

In the three already identified classes of canonical EVH1 domains belonging to the Ena/Vasp/Homer/Spred families [33,34] a conserved phenylalanine and tryptophan are essential for the formation of the recognition surface that accommodates the proline-rich ligand. We discovered that in Falafel the conserved phenylalanine is replaced by leucine and experimentally proved that this residue is essential for binding to FxxP and MxPP motif-containing target proteins. Moreover, we demonstrated that this leucine is invariant in all EVH1 domains of the PP4R3 orthologues, from yeast to humans. Accordingly, mutation of Leu70 (in fruit fly Falafel) or Leu69 (in human SMEK1) to alanine altered the interaction with the target proteins. These findings reveal a novel mode of PP4 binding, in which a conserved Leu is essential. They further suggest that the EVH1 domain of PP4 may represent a new type (or class) within the EVH1 family that is able to accommodate low proline content sequences, such as the FxxP motif. Given the increased flexibility offered by PP4R3 orthologues that use leucine instead of phenylalanine it is unclear why the former is not more prevalent. It will be a future challenge to unveil the background for this evolutional selection.

Finally, our work sheds light on the function of the Smk-1 domain of Falafel. Surprisingly, although Smk-1 is present in human SMEK1 and SMEK2, too, its function was not described in the recent work by Ueki and colleagues [32]. Indeed, it is not clear whether Smk-1 represents an independent domain or is just part of the ARM/Heat repeats occupying the middle regions of R3 orthologues. Nevertheless, it has been shown that the Smk-1 domain of mammalian SMEK1 physically interacts with Par3 in neural progenitor cells [29], suggesting that PP4R3 uses at least two different substrate-binding mechanisms through its conserved domains, EVH1 and Smk-1. Our findings unequivocally support this model. We identified several Smk-1 interacting proteins in *Drosophila*, including components of the spindle assembly checkpoint, a process that is believed to be regulated by PP4. We showed that Smk-1 binds to its targets in an FxxP- and MxPP-independent manner, however, we were not able to identify the regions specific to Smk-1. It would be important to identify more binding partners of Smk-1 in flies or in mammalian models by AP-MS or other techniques, and clarify the crystal structure and mode of binding of this domain. This would contribute to a better understanding of how the Smk-1-mediated target-binding of PP4 works and why PP4R3 orthologues have different binding mechanism.

One may assume that the two target-binding domains with different structure in a single regulatory subunit allow the PP4c-R2-R3 holoenzyme to recognize and thus regulate different types of molecules in different biological processes and in various tissues. Having two independent domains in a single protein can give maximum flexibility but also maximum stringency to the enzyme. Future studies are required to investigate this hypothesis in detail.

## 4. Material and methods

### 4.1. DNA constructs and cloning

Complementary DNA (cDNA) encoding *Drosophila* proteins (electronic supplementary material, table S2) were obtained from the *Drosophila* Genomics Resource Center (DGRC), while human SMEK1/PP4R3*α* cDNA (Clone Id: 6142109) was purchased from the Mammalian Gene Collection (Horizon). To generate entry clones, human SMEK1^EVH1^ (Hs-EVH1 (aa 1–168)) or *Drosophila* Zw10 and Zwilch were respectively cloned into the pDONR221 vector using the Gateway Cloning System (Thermo Fisher Scientific). Entry clones of Falafel domains (Flfl^1-168aa^ (EVH1) and Flfl^169-361aa^ (Smk-1)) were previously described [8]. Entry clones containing Dm-EVH1^L70A^, Hs-EVH1^L69A^, Zw10-^238^AxxA^241^ or Zw10-^438^AxxA^441^ were created according to standard procedures using the QuikChange II XL Site-Directed Mutagenesis Kit (Agilent Technologies). Expression constructs were made by LR reaction using the following destination vectors: pUGW (N-terminal eGFP fusion under the regulation of the constitutively active *polyubiquitin* promoter in D.Mel-2 cultured cells (*Drosophila* Gateway Vector Collection, DGVC)), pAFW (N-terminal 3xFLAG fusion under the regulation of the constitutively active *actin5c* promoter in D.Mel-2 cells, DGVC), pF3A_3xFlag-GW (N-terminal 3xFLAG fusion under the regulation of the SP6 promoter for wheat germ-based *in vitro* expression system, in house modified pF3A-WG (Promega)) and pDEST15 (N-terminal GST fusion in *Escherichia coli*, Thermo Fisher Scientific). *Drosophila* cDNAs in pOT2 vector or cDNAs sub-cloned into the pHY22 plasmid were used directly in the T7 promoter-based IVTT expression system to generate ^35^S-methionine labelled untagged proteins (electronic supplementary material, table S2). AxxA and AxPA mutations were generated in cDNAs (in pOT2 or pHY22 plasmids) using the QuikChange II XL Site-Directed Mutagenesis Kit. Linear DNA fragments encoding overlapping segments of the prey proteins (electronic supplementary material, figures S4 and S6) were generated by PCR to create the following IVTT-compatible configuration: T7 promoter-Kozak sequence-ATG-gene-specific sequence-STOP codon. Oligonucleotide primers are listed in electronic supplementary material, table S3.

### 4.2. Recombinant protein expression and purification

GST, GST-EVH1, GST-Dm-EVH1^L70A^, GST-Smk-1, GST-Hs-EVH1 and GST-Hs-EVH1^L69A^ were expressed in *E. coli* SixPack strain [52] and purified to homogeneity as follows. Cells were grown in 50 ml LB medium to A_600_ approximately 0.6 and expression was induced with 0.5 mM isopropyl 1-thio-β-D-galactopyranoside for 5 h at 25°C. Cells were lysed by ultrasound disruption in 30 ml phosphate buffered saline (PBS) supplemented with 1 mM PMSF and 0.2 mg ml^−1^ lysozyme, followed by centrifugation at 16 000×*g* at 4°C for 15 min. The cleared supernatant was loaded onto pre-equilibrated glutathione sepharose 4B resin (GE Healthcare) and incubated at 4°C for 1.5 h. Then, beads were washed five times with PBS supplemented with 0.05% Triton X-100, immobilized proteins were kept on beads and stored at −20°C in PBS supplemented with 50% glycerol.

### 4.3. Affinity-purification coupled to mass spectrometry

For MS identification of EVH1- and Smk-1-interacting proteins, whole cell protein extracts in EB buffer (50 mM HEPES pH 7.5, 100 mM CH3COOK, 100 mM NaCl, 50 mM KCl, 2 mM MgCl_2_, 2 mM EGTA-Na, 5 mM DTT, 0.5% NP-40, 5% glycerol and complete protease inhibitor cocktail (Merck)) were generated from 3 g of wild-type (*white^1118^*) syncytial embryos [53]. Equal amounts of clarified lysates were mixed with GST, GST-EVH1 or GST-Smk-1 immobilized onto beads (electronic supplementary material, figure S1A), respectively, incubated at 4°C for 2 h, washed several times with EB buffer and once in PBS. Bound proteins were analysed by mass spectrometry after on-beads digestion with trypsin according to standard procedures [53,54].

### 4.4. IVTT and *in vitro* binding assays

For *in vitro* PPI experiments ^35^S-methionine-labelled prey proteins (full-length wild-type or mutant and truncated forms) were produced *in vitro* using TnT T7 Quick IVTT (Promega, L1170). Fifty nanograms of purified PCR products or recombinant plasmids were added to a 15 µl reaction, containing TnT Quick Master Mix, RNasin Plus RNase Inhibitor, T7 TnT PCR Enhancer, EDTA-free protease inhibitor cocktail (Merck) and 0.3 MBq Methionine-L [^35^S] (Perkin Elmer). After incubating the mixture at 30°C for 1 h, samples were centrifuged at 12 000×*g* at 25°C for 5 min. The supernatant (IVTT input) was divided into equal parts and used for PPI experiments in which GST served as a negative control and GST-fused proteins were used as bait. GST or GST-fused bait proteins were immobilized on glutathione sepharose 4B resin (GE Healthcare), equilibrated with 1 ml WB1 (50 mM HEPES pH 7.5, 150 mM NaCl, 2 mM MgCl_2_, 1 mM EGTA, 1 mM DTT, 0.1% Triton X-100), beads were settled by centrifugation (600×*g*, 4°C for 3 min), re-suspended in 800 µl of binding buffer (WB1 supplemented with EDTA-free protease inhibitor cocktail (Merck) and 0.5 mg ml^−1^ BSA), mixed with an equal amount of ^35^S-methionine-labelled prey proteins and incubated for 90 min at 4°C. Beads were washed three times with WB1 and three times with WB2 (WB1 supplemented with 50 mM NaCl and 0.1% Triton X-100), transferred to new tubes and boiled in 10 µl Laemmli sample buffer. Proteins (5% IVTT input and 100% bound) were separated by SDS-PAGE. Resultant gels were stained with Coomassie Brilliant Blue, scanned, dried and directly used for autoradiography. Exposure to autoradiography film (Kodak) was carried out at −80°C.

Flag-ROD (from pF3A_3xFLAG-ROD plasmid) was expressed in TnT SP6 High-Yield Wheat Germ Protein Expression System according to the manufacturer (Promega), separated by SDS-PAGE, blotted to nitrocellulose (Amersham) and visualized by immunoblotting using an anti-FlagM_2_ antibody.

### 4.5. Co-Immunoprecipitation from D.Mel-2 cells

D.Mel-2 cells (Life Technologies) were grown in Insectagro DS2 serum-free medium (Corning) supplemented with 2 mM stable-glutamine (Biosera) and PenStrep (Gibco). Cells were transiently co-transfected using Cellfectin II reagent (Thermo Fisher Scientific) with GFP or GFP-Smk-1 (in pUGW plasmid) and Flag-Zw10 or Flag-Zwilch (in pAFW plasmid, wild-type or mutant), respectively and collected 48 h post-transfection. Cells were lysed in 500 µl EB buffer (supplemented with 25 µM MG132 and 0.1 µl ml^−1^ Benzonase Nuclease) by passing the cell suspension through a G25 needle (ten times) followed by centrifugation at 16 000 × *g* at 4°C for 10 min. The supernatants were incubated with GFP-Trap magnetic agarose beads (Chromotek) at 4°C for 90 min. The beads were washed five times with EB buffer and boiled in Laemmli sample buffer. Inputs and bound proteins were separated by SDS-PAGE, transferred to polyvinylidene fluoride (PVDF) membrane and analysed by immunoblotting using anti-GFP and anti-FlagM_2_ antibodies.

### 4.6. Antibodies

For the immunoblotting the following antibodies were used: mouse anti-GFP (1 : 10 000; Abcam, ab190584), mouse anti-FlagM_2_ (1 : 10 000; Sigma F1804) and polyclonal goat anti-mouse IgG/HRP (1 : 10 000; DAKO, P0447).

### 4.7. Predictions and alignments

Intrinsically disordered protein regions were predicted using the IUPred2A online software (https://iupred2a.elte.hu/) [55]. Protein domains were predicted using InterPro (https://www.ebi.ac.uk/interpro/) [56]. Multiple sequence alignments were done with Clustal Omega at EMBL-EBI (https://www.ebi.ac.uk/Tools/msa/clustalo/) [57]. Protein sequences were retrieved from Uniprot (https://www.uniprot.org/) [58].

## Supplementary Material

Supplemental material

## Data accessibility

The raw data are available from the authors.

## References

[1] Brautigan DL 2013 Protein Ser/Thr phosphatases–the ugly ducklings of cell signalling. FEBS J. 280, 324-345. (10.1111/j.1742-4658.2012.08609.x) 22519956

[2] Manning G, Whyte DB, Martinez R, Hunter T, Sudarsanam S 2002 The protein kinase complement of the human genome. Science 298, 1912–1934. (10.1126/science.1075762) 12471243

[3] Moura M, Conde C 2019 Phosphatases in mitosis: roles and regulation. Biomolecules 9, 55 (10.3390/biom9020055) PMC6406801 30736436

[4] Virshup DM, Shenolikar S 2009 From promiscuity to precision: protein phosphatases get a makeover. Mol. Cell 33, 537–545. (10.1016/j.molcel.2009.02.015) 19285938

[5] Park J, Lee DH 2020 Functional roles of protein phosphatase 4 in multiple aspects of cellular physiology: a friend and a foe. BMB Rep. 53, 181–190. (10.5483/BMBRep.2020.53.4.019) 32192570 PMC7196183

[6] Barr FA, Elliott PR, Gruneberg U 2011 Protein phosphatases and the regulation of mitosis. J. Cell Sci. 124, 2323–2334. (10.1242/jcs.087106) 21709074

[7] Chen F et al*.* 2007 Multiple protein phosphatases are required for mitosis in *Drosophila*. Curr. Biol. 17, 293–303. (10.1016/j.cub.2007.01.068) 17306545

[8] Lipinszki Z, Lefevre S, Savoian MS, Singleton MR, Glover DM, Przewloka MR 2015 Centromeric binding and activity of protein phosphatase 4. Nat. Commun. 6, 5894 (10.1038/ncomms6894) 25562660 PMC4354016

[9] Helps NR, Brewis ND, Lineruth K, Davis T, Kaiser K, Cohen PTW 1998 Protein phosphatase 4 is an essential enzyme required for organisation of microtubules at centrosomes in *Drosophila* embryos. J. Cell Sci. 111, 1331–1340. 9570751 10.1242/jcs.111.10.1331

[10] Zhuang X, Semenova E, Maric D, Craigie R 2014 Dephosphorylation of barrier-to-autointegration factor by protein phosphatase 4 and its role in cell mitosis. J. Biol. Chem. 289, 1119–1127. (10.1074/jbc.M113.492777) 24265311 PMC3887179

[11] Toyo-oka K et al*.* 2008 Protein phosphatase 4 catalytic subunit regulates Cdk1 activity and microtubule organization via NDEL1 dephosphorylation. J. Cell Biol. 180, 1133–1147. (10.1083/jcb.200705148) 18347064 PMC2290842

[12] Torras-Llort M, Medina-Giro S, Escudero-Ferruz P, Lipinszki Z, Moreno-Moreno O, Karman Z, Przewloka MR, Azorin F 2020 A fraction of barrier-to-autointegration factor (BAF) associates with centromeres and controls mitosis progression. Commun. Biol. 3, 454 (10.1038/s42003-020-01182-y) 32814801 PMC7438335

[13] Lee DH, Pan Y, Kanner S, Sung P, Borowiec JA, Chowdhury D 2010 A PP4 phosphatase complex dephosphorylates RPA2 to facilitate DNA repair via homologous recombination. Nat. Struct. Mol. Biol. 17, 365–372. (10.1038/nsmb.1769) 20154705 PMC3057140

[14] O'Neill BM, Szyjka SJ, Lis ET, Bailey AO, Yates JR 3rd, Aparicio OM, Romesberg FE 2007 Pph3–Psy2 is a phosphatase complex required for Rad53 dephosphorylation and replication fork restart during recovery from DNA damage. Proc. Natl Acad. Sci. USA 104, 9290–9295. (10.1073/pnas.0703252104) 17517611 PMC1890487

[15] Keogh MC et al. 2006 A phosphatase complex that dephosphorylates γH2AX regulates DNA damage checkpoint recovery. Nature 439, 497–501. (10.1038/nature04384) 16299494

[16] Lee DH et al*.* 2014 Dephosphorylation enables the recruitment of 53BP1 to double-strand DNA breaks. Mol. Cell 54, 512–525. (10.1016/j.molcel.2014.03.020) 24703952 PMC4030556

[17] Lee DH, Goodarzi AA, Adelmant GO, Pan Y, Jeggo PA, Marto JA, Chowdhury D 2012 Phosphoproteomic analysis reveals that PP4 dephosphorylates KAP-1 impacting the DNA damage response. EMBO J. 31, 2403–2415. (10.1038/emboj.2012.86) 22491012 PMC3364739

[18] Shaltiel IA, Aprelia M, Saurin AT, Chowdhury D, Kops GJ, Voest EE, Medema RH 2014 Distinct phosphatases antagonize the p53 response in different phases of the cell cycle. Proc. Natl Acad. Sci. USA 111, 7313–7318. (10.1073/pnas.1322021111) 24711418 PMC4034242

[19] Carnegie GK, Sleeman JE, Morrice N, Hastie CJ, Peggie MW, Philp A, Lamond AI, Cohen PT 2003 Protein phosphatase 4 interacts with the survival of motor neurons complex and enhances the temporal localisation of snRNPs. J. Cell Sci. 116, 1905–1913. (10.1242/jcs.00409) 12668731

[20] Kim BR, Kwon Y, Rho SB 2017 BMI-1 interacts with sMEK1 and inactivates sMEK1-induced apoptotic cell death. Oncol. Rep. 37, 579–586. (10.3892/or.2016.5262) 27878292

[21] Moon BS, Yun HM, Chang WH, Steele BH, Cai M, Choi SH, Lu W 2017 Smek promotes corticogenesis through regulating Mbd3's stability and Mbd3/NuRD complex recruitment to genes associated with neurogenesis. PLoS Biol. 15, e2001220 (10.1371/journal.pbio.2001220) 28467410 PMC5414985

[22] Sousa-Nunes R, Chia W, Somers WG 2009 Protein phosphatase 4 mediates localization of the Miranda complex during *Drosophila* neuroblast asymmetric divisions. Genes Dev. 23, 359–372. (10.1101/gad.1723609) 19204120 PMC2648543

[23] Cohen PT, Philp A, Vazquez-Martin C 2005 Protein phosphatase 4—from obscurity to vital functions. FEBS Lett. 579, 3278–3286. (10.1016/j.febslet.2005.04.070) 15913612

[24] Gingras AC et al. 2005 A novel, evolutionarily conserved protein phosphatase complex involved in cisplatin sensitivity. Mol. Cell. Proteomics: MCP. 4, 1725–1740. (10.1074/mcp.M500231-MCP200) 16085932

[25] Hwang J, Lee JA, Pallas DC 2016 Leucine carboxyl methyltransferase 1 (LCMT-1) methylates protein phosphatase 4 (PP4) and protein phosphatase 6 (PP6) and differentially regulates the stable formation of different PP4 holoenzymes. J. Biol. Chem. 291, 21 008–21 019. (10.1074/jbc.M116.739920) PMC5076511 27507813

[26] Ho Y et al. 2002 Systematic identification of protein complexes in *Saccharomyces cerevisiae* by mass spectrometry. Nature 415, 180–183. (10.1038/415180a) 11805837

[27] Gavin AC et al*.* 2002 Functional organization of the yeast proteome by systematic analysis of protein complexes. Nature 415, 141–147. (10.1038/415141a) 11805826

[28] Zarske M, Hafen E 2003 Falafel, a novel EVH1 domain protein involved in Rac mediated epithelial morphogenesis. In 44th Annual Drosophila Research Conference, Chicago, March 5–9, 2003 Bethesda, MD: The Genetics Society of America.

[29] Lyu J, Kim HR, Yamamoto V, Choi SH, Wei Z, Joo CK, Lu W 2013 Protein phosphatase 4 and Smek complex negatively regulate Par3 and promote neuronal differentiation of neural stem/progenitor cells. Cell Rep. 5, 593–600. (10.1016/j.celrep.2013.09.034) 24209749 PMC3855259

[30] Ma H, Han BK, Guaderrama M, Aslanian A, Yates JR 3rd, Hunter T, Wittenberg C 2014 Psy2 targets the PP4 family phosphatase Pph3 to dephosphorylate Mth1 and repress glucose transporter gene expression. Mol. Cell. Biol. 34, 452–463. (10.1128/MCB.00279-13) 24277933 PMC3911506

[31] Wolff S, Ma H, Burch D, Maciel GA, Hunter T, Dillin A 2006 SMK-1, an essential regulator of DAF-16-mediated longevity. Cell 124, 1039–1053. (10.1016/j.cell.2005.12.042) 16530049

[32] Ueki Y et al*.* 2019 A consensus binding motif for the PP4 protein phosphatase. Mol. Cell. 76, 953–964. (10.1016/j.molcel.2019.08.029) 31585692 PMC6981294

[33] Ball LJ, Jarchau T, Oschkinat H, Walter U 2002 EVH1 domains: structure, function and interactions. FEBS Lett. 513, 45–52. (10.1016/s0014-5793(01)03291-4) 11911879

[34] Peterson FC, Volkman BF 2009 Diversity of polyproline recognition by EVH1 domains. Front. Biosci. (Landmark edition). 14, 833–846. (10.2741/3281) PMC3882067 19273103

[35] Voss M et al 2013 Protein phosphatase 4 is phosphorylated and inactivated by Cdk in response to spindle toxins and interacts with gamma-tubulin. Cell Cycle 12, 2876–2887. (10.4161/cc.25919) 23966160 PMC3899200

[36] van der Waal MS, Hengeveld RC, van der Horst A, Lens SM. 2012 Cell division control by the chromosomal passenger complex. Exp. Cell Res. 318, 1407–1420. (10.1016/j.yexcr.2012.03.015) 22472345

[37] Carmena M, Wheelock M, Funabiki H, Earnshaw WC 2012 The chromosomal passenger complex (CPC): from easy rider to the godfather of mitosis. Nat. Rev. Mol. Cell Biol. 13, 789–803. (10.1038/nrm3474) 23175282 PMC3729939

[38] Tovey CA, Conduit PT 2018 Microtubule nucleation by γ-tubulin complexes and beyond. Essays Biochem. 62, 765–780. (10.1042/EBC20180028) 30315097 PMC6281477

[39] Karess R 2005 Rod-Zw10-Zwilch: a key player in the spindle checkpoint. Trends Cell Biol. 15, 386–392. (10.1016/j.tcb.2005.05.003) 15922598

[40] Barbosa J, Conde C, Sunkel C 2020 RZZ-SPINDLY-DYNEIN: you got to keep ‘em separated. Cell Cycle. 19, 1716–1726. (10.1080/15384101.2020.1780382) 32544383 PMC7469663

[41] Prehoda KE, Lee DJ, Lim WA 1999 Structure of the enabled/VASP homology 1 domain-peptide complex: a key component in the spatial control of actin assembly. Cell 97, 471–480. (10.1016/s0092-8674(00)80757-6) 10338211

[42] McHugh DR, Koumis E, Jacob P, Goldfarb J, Schlaubitz-Garcia M, Bennani S, Regan P, Patel P, Youngman MJ 2020 DAF-16 and SMK-1 contribute to innate immunity during adulthood in *Caenorhabditis elegans*. G3 10, 1521–1539. (10.1534/g3.120.401166) 32161087 PMC7202018

[43] Ehrlich P 1960 *The collected papers of Paul Ehrlich: Chemotherapy* London, UK: Pergamon Press.

[44] Egloff MP, Johnson DF, Moorhead G, Cohen PT, Cohen P, Barford D 1997 Structural basis for the recognition of regulatory subunits by the catalytic subunit of protein phosphatase 1. EMBO J. 16, 1876–1887. (10.1093/emboj/16.8.1876) 9155014 PMC1169791

[45] Terrak M, Kerff F, Langsetmo K, Tao T, Dominguez R 2004 Structural basis of protein phosphatase 1 regulation. Nature 429, 780–784. (10.1038/nature02582) 15164081

[46] Hendrickx A, Beullens M, Ceulemans H, Den Abt T, Van Eynde A, Nicolaescu E, Lesage B, Bollen M. 2009 Docking motif-guided mapping of the interactome of protein phosphatase-1. Chem. Biol. 16, 365–371. (10.1016/j.chembiol.2009.02.012) 19389623

[47] Roy J, Li H, Hogan PG, Cyert MS 2007 A conserved docking site modulates substrate affinity for calcineurin, signaling output, and in vivo function. Mol. Cell 25, 889–901. (10.1016/j.molcel.2007.02.014) 17386265 PMC2913616

[48] Gal M, Li S, Luna RE, Takeuchi K, Wagner G 2014 The LxVP and PxIxIT NFAT motifs bind jointly to overlapping epitopes on calcineurin's catalytic domain distant to the regulatory domain. Structure 22, 1016–1027. (10.1016/j.str.2014.05.006) 24954618 PMC4102887

[49] Wang X, Bajaj R, Bollen M, Peti W, Page R 2016 Expanding the PP2A interactome by defining a B56-specific SLiM. Structure 24, 2174–2181. (10.1016/j.str.2016.09.010) 27998540 PMC5180209

[50] Hertz EPT, Kruse T, Davey NE, Lopez-Mendez B, Sigurethsson JO, Montoya G, Olsen JV, Nilsson J 2016 A conserved motif provides binding specificity to the PP2A-B56 phosphatase. Mol. Cell. 63, 686–695. (10.1016/j.molcel.2016.06.024) 27453045

[51] Cundell MJ, Hutter LH, Nunes Bastos R, Poser E, Holder J, Mohammed S, Novak B, Barr FA 2016 A PP2A-B55 recognition signal controls substrate dephosphorylation kinetics during mitotic exit. J. Cell Biol. 214, 539–554. (10.1083/jcb.201606033) 27551054 PMC5004449

[52] Lipinszki Z, Vernyik V, Farago N, Sari T, Puskas LG, Blattner FR, Posfai G, Gyorfy Z 2018 Enhancing the translational capacity of *E. coli* by resolving the codon bias. ACS Synth. Biol. 7, 2656–2664. (10.1021/acssynbio.8b00332) 30351909

[53] Lipinszki Z, Wang P, Grant R, Lindon C, Dzhindzhev NS, D'Avino PP, Przewloka MR, Glover DM, Archambault V 2014 Affinity purification of protein complexes from *Drosophila* embryos in cell cycle studies. Methods Mol. Biol. 1170, 571–588. (10.1007/978-1-4939-0888-2_33) 24906338

[54] Haider S, Lipinszki Z, Przewloka MR, Ladak Y, D'Avino PP, Kimata Y, Lio P, Glover DM 2015 DAPPER: a data-mining resource for protein–protein interactions. BioData Mining 8, 30 (10.1186/s13040-015-0063-3) 26405458 PMC4581157

[55] Mészáros B, Erdos G, Dosztányi Z 2018 IUPred2A: context-dependent prediction of protein disorder as a function of redox state and protein binding. Nucleic Acids Res. 46, W329–W337. (10.1093/nar/gky384) 29860432 PMC6030935

[56] Mitchell AL et al 2019 InterPro in 2019: improving coverage, classification and access to protein sequence annotations. Nucleic Acids Res. 47, D351–D360. (10.1093/nar/gky1100) 30398656 PMC6323941

[57] Goujon M, McWilliam H, Li W, Valentin F, Squizzato S, Paern J, Lopez R 2010 A new bioinformatics analysis tools framework at EMBL–EBI. Nucleic Acids Res. 38, W695–W699. (10.1093/nar/gkq313) 20439314 PMC2896090

[58] Consortium TU 2018 UniProt: a worldwide hub of protein knowledge. Nucleic Acids Res. 47, D506–D515. (10.1093/nar/gky1049) PMC6323992 30395287

