# Supplementary material for: Novel perspectives of target-binding by the evolutionarily conserved PP4 phosphatase
Source: Open Biol. 2020 Dec 23;10(12):200343. doi: 10.1098/rsob.200343 (PMC7776573; doi:10.1098/rsob.200343)
Supplement: Supplemental material [file rsob200343supp1.pdf]

Supplemental material

Novel perspectives of target-binding by the evolutionarily conserved PP4 phosphatase

Zoltan Karman[1,2], Zsuzsanna Rethi-Nagy[1,2], Edit Abraham[1], Lilla Fabri-Ordogh[1], Akos Csonka[3], Peter Vilmos[4], Janusz Debski[5], Michal Dadlez[5], David M. Glover[6] and Zoltan Lipinszki[1]

[1]Biological Research Centre, Institute of Biochemistry, MTA Lendület Laboratory of Cell Cycle Regulation, Szeged, Hungary
[2]Doctoral School of Biology, Faculty of Science and Informatics, University of Szeged, Szeged, Hungary
[3]Department of Traumatology, University of Szeged, Szeged, Hungary
[4]Biological Research Centre, Institute of Genetics, Szeged, Hungary
[5]Laboratory of Mass Spectrometry, Institute of Biochemistry and Biophysics, Polish Academy of Sciences, 02-106 Warsaw, Poland
[5]Department of Genetics, University of Cambridge, Cambridge CB2 3EH, UK

Author for correspondence:
Zoltan Lipinszki
e-mail: lipinszki.zoltan@brc.hu

Figures

Figure S1

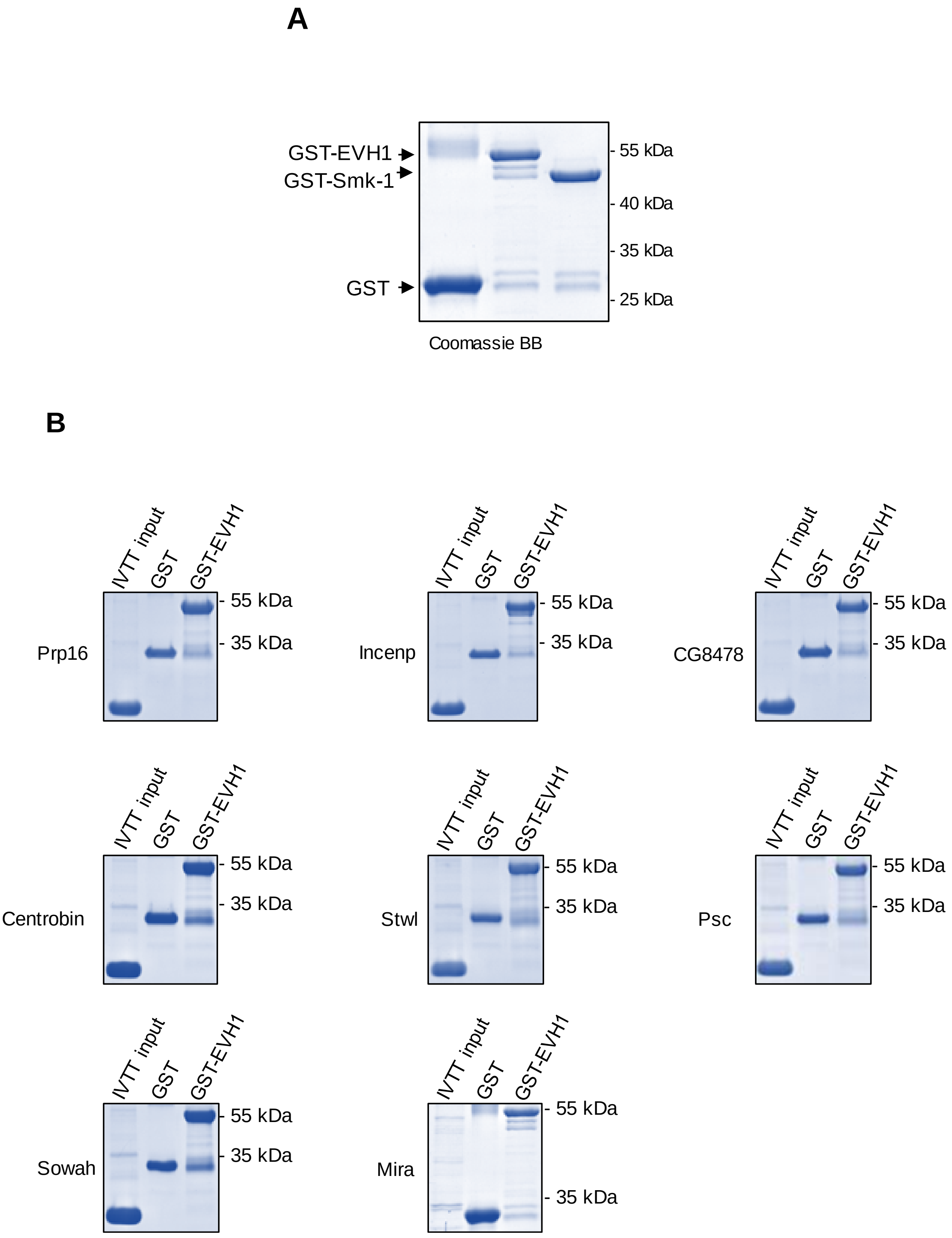

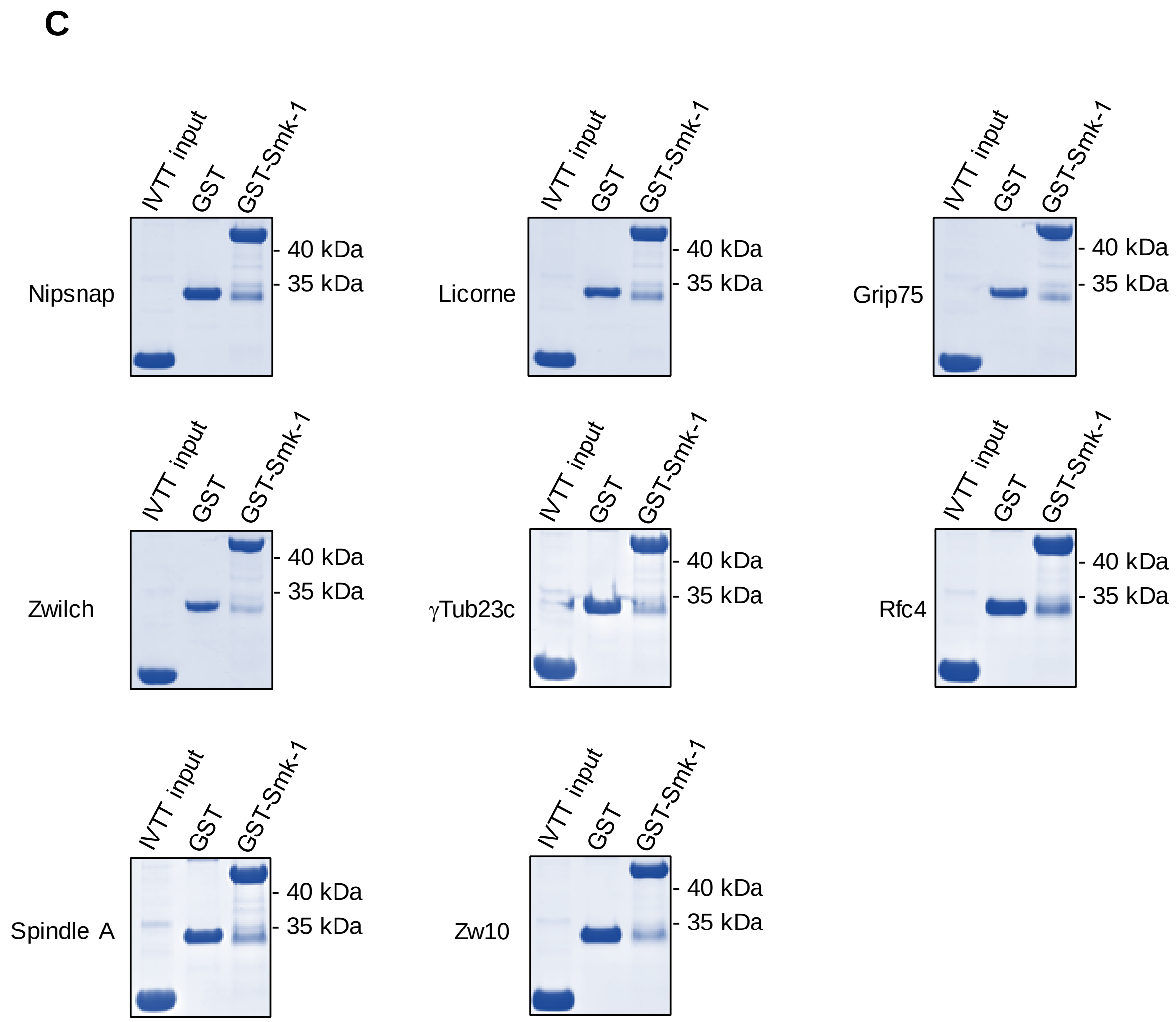
C
IVTT input
GST
GST-Smk-1
40 kDa
35 kDa
Nipsnap
Licorne
Grip75
Zwilch
γTub23c
Rfc4
Spindle A
Zw10

D

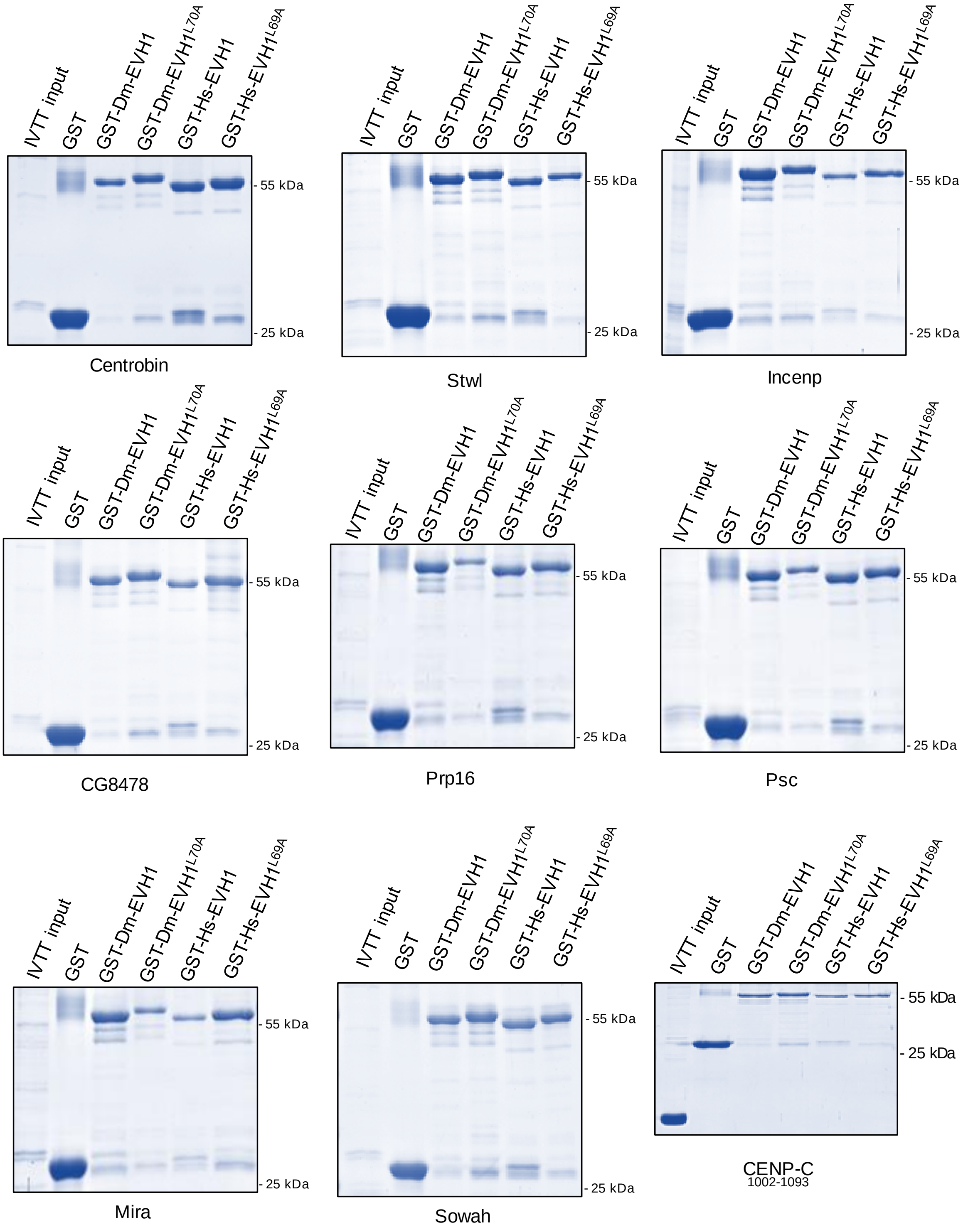

Figure S1. Amounts of bait proteins used in AP-MS and protein-protein interaction assays. A. GST, GST-EVH1 or GST-Smk-1 were purified from *E. coli* and immobilised onto Glutathione Sepharose 4B beads. A Coomassie Brilliant Blue-stained SDS-PAGE gel shows that equal amounts of proteins were used in AP-MS as well as

protein-protein interaction assays. **B**. Coomassie Brilliant Blue-stained SDS-PAGE gels corresponding to autoradiographs shown in Figure 2A were scanned before drying and autoradiography. Gels show the amount of bait (GST and GST-EVH1) proteins used in *in vitro* binding assay. **C.** Coomassie Brilliant Blue-stained SDS-PAGE gels corresponding to autoradiographs shown in Figure 5A were scanned before drying and autoradiography. Gels show the amount of bait (GST and GST-Smk-1) proteins used in *in vitro* binding assay. The abundant protein present in the IVTT inputs is globin from the reticulocyte lysate. **D.** Coomassie Brilliant Blue-stained SDS-PAGE gels corresponding to autoradiographs shown in Figure 4B were scanned before drying and autoradiography. Gels show the amount of bait (GST, GST-Dm-EVH1, GST-Dm-EVH1^{L70A}, GST-Hs-EVH1 and GST-Hs-EVH1^{L69A}) proteins used in *in vitro* binding assay.

Figure S2

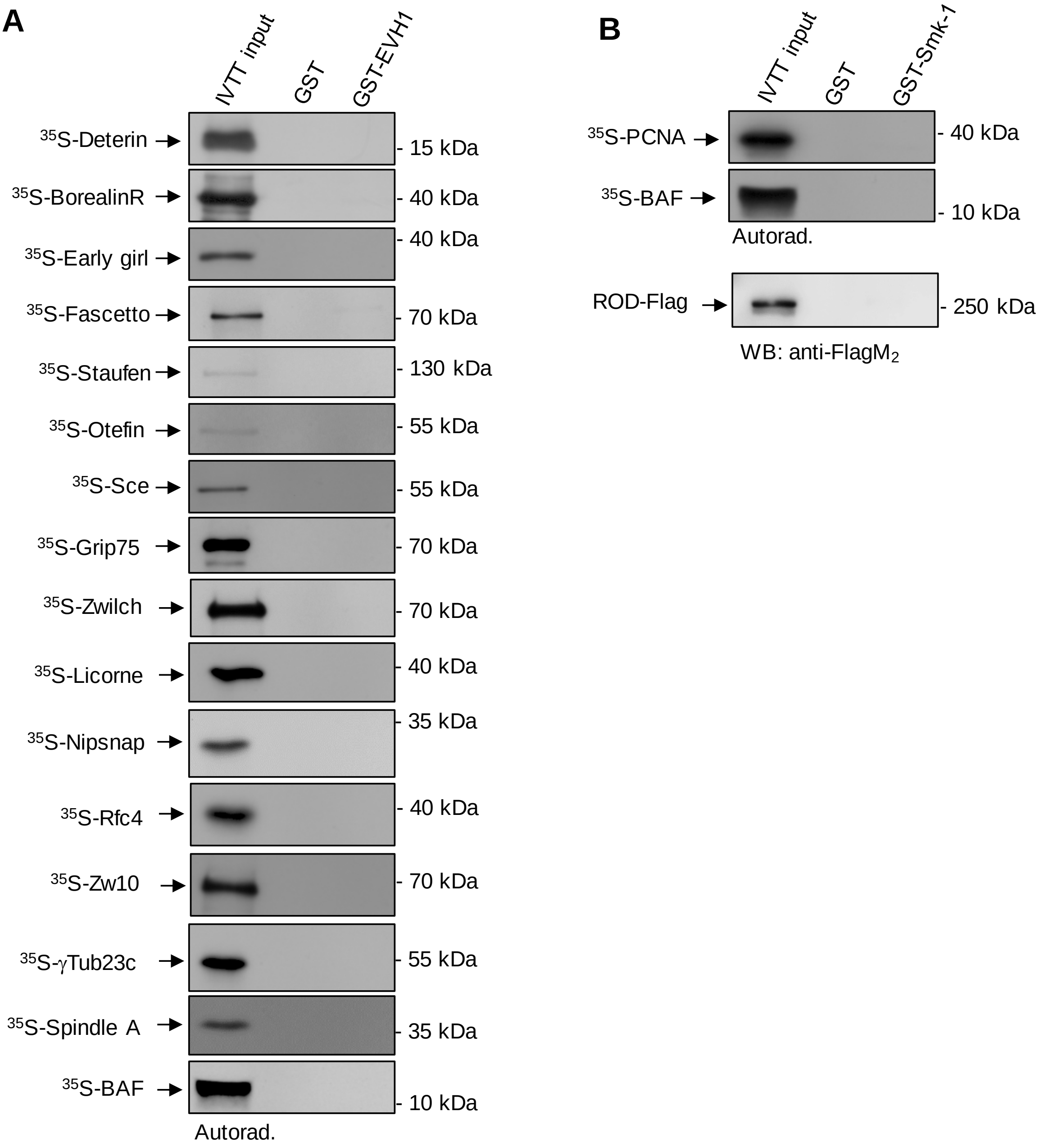

Figure S2. Several AP-MS-identified proteins do not show interaction with EVH1 or Smk-1. A. Autoradiographs presenting that there is no physical interaction between GST-tagged EVH1 and the indicated IVTT-produced ^{35}S-methionine-labelled prey proteins. **B**. Autoradiograph and Western-blot (anti-FlagM$_2$) show that there is no physical interaction between GST-tagged Smk-1 and the indicated IVTT-produced ^{35}S-methionine-labelled or IVTT (wheat germ)-produced Flag-tagged prey proteins. GST served as negative control.

Figure S3

<u>A. EVH1-interacting proteins:</u>

>**Incenp** (A0A0B4LFQ2)
MEDILGVLGSLSDLRRELEVLRKAHFEELDHLFYGTGQPEAEAKPRDSAPAAKSQENSSVTPQQTKKRPKRLTSLAEDQ
NEPEAPDATANNTSARQSTRVSNSQLLAIAEDEHNSTASL**<u>MPPP</u>**PVPVSADTTLGSGRPQRAAKLKTEKLLKEPSLNR
KMRRPSSEELVKVKVESEQRVSQFNSFTSAQALEENKLAEPELEEPAAETAEQQKPPEEASVTEDVNTTKTLRVKVKRE
KLSTEAVPPLTNAVSTANVTTVSSVTTEAARPDDTVASNTTTSTEVSKKVRKKKKDVESHRPIKVERFSDLDKSSPVSSR
TRKCSSDSRTVQERSIYKDALEDPPVAEQSVAAVAPAAVNETVAISNATLVLGPAPTSDSPIGPAGDATFEVHSNDKK
QPLSKQDSLLTEDESVEEKLPTTKLLSSIKAIKLPTRTHELFNPLLQSPVKMRVEAFENAANAQNSMRPKRGKDLQGTP
GSNNTPKIGKLPAPTVGR**<u>FFTP</u>**TQTTSTLPLSSAQPKKGPASASKATSLLKTATGTNLRSVNSTSTKTLSRENSGDDFRK
GLHNLAEERKKLREQKHQQAAQQREAKERERAERMAKLAAERAKKQEERKRIEERKRQELEELQRKMRQQEEAEAL
KKAKFKELEQQKLQQLTGAKPKK**<u>MLPP</u>**PPKTKYTWEMLHEDDSTDDEGKVTHKRPPAPTWSRSHVRGEAIAMQSH
CPTDIIDSFFSVAPTTPDLKQIFPNIDPSQLKRNSSVLWSTPPRYSELPKY

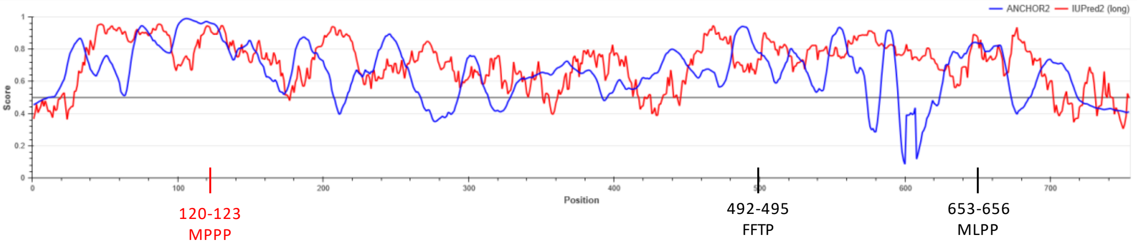

>**Posterior sex combs** (P35820)
MMTPESKAIQPAAATTKQTAEATATTTMAHTQQKSQLSTLAKTTTTTATNKAAKSVVSNANSSGNNSSKKLALSQS
QKTTTTTTPPTTTTTTTAAAAAEATTNADKMQKQQQLKQQLFAACSIKVKSENTLATTANAALAAATTTTTTATPALA
TGKAAKTILENGIKKESTPPAVESVEASSSSSSSSSSSSSSSSSSWPTTRRATSEDASSNGGASADEEKSEEDPTAAVAASS
TATTTSDLATTSRPRPVLLTAVNPHIICHLCQGYLINATTIVECLHSFCHSCLINHLRKERFCPRCEMVINNAKPNIKSDTT
LQAIVYKLVPGLYERELMRKRAFYKDRPEEAALATPEQRGDDTEHLIFSPSDDMSLSLEYAELGELKTDSEPELVDTLRP
RYLQCPAMCRVSHLKKFVYDKFEIDAQRFSIDIMYKVKTIVLLDYYTLMDIAYIYTWKRDAPMRFYYRVYESPQPLVKP
APRRVLPLKLEKQERENQEQQLAVEVASSKVEPVSLPEDQKAEASIKVEEQESTREIVKEVIKDVAATPPTETLKLVINR
NMLDKREKSHSPQMSSKSSSKSSPCTPVSSPSEPNIKLKIDLSKQNSVTIIDMSDPERREIVKPLKPEKESRSKKKDKDGS
PKSSSSSSSSSSGERKRKSPSPLTVPPLTIRTERIMSPSGVSTLSPRVTSGAFSEDPKSEFLKSFALKPIKVKVESPERTLNNR
AITPPSPSVQQSASPKSKGNNLDDSIL**<u>MKPP</u>**SCMPPKSIASSKRKSKEPVKAVSKKQKLSPPLPTVDFKIRLPVTNGNSS
GTASPKIEKPL**<u>MPPP</u>**AKPPMLAPRKLQPSAQ**<u>FAPP</u>**PSPIHHHAGVQMSAPGNRTPIAKRYQPILPKASRPNPFANIPN
DVNRLLKDAGTEIKSIGGGSVENNSNAAQKPHLYGPKGESKMGPPALPATTPSQGNKNVGKQAGNLPMSAPPNKG
NSSNNYLNLALFNSSKCKGKEAPPGCRTPMYTPNSPIYSPSSPQYVPSYNIPTMPTYKYTPKPTPNSGSGNGGSGSYLQ
NMLGGGNGGSLGGL**<u>FPSP</u>**PTKSDQNTNPAQGGGGSSSATQSGGNNGIVNNNIYMPNEDAPEKQQVKVKSLLNSC
NINIPSSLSITISRDNGDSSSPNNGQHPKHKSPVNNYIEIVKLPDQPQDQVQAAKEAQKRQSPPAAVPGHLAAKLPPPP
PSKAIPSPQHLVSR**<u>MTPP</u>**QLPKVATPPPPSSPRVITPPKTSPPANAAKVTPLKPVLTPTQVDKKTPSPEKRTAAQMGSH
SPTASENKSPKGGPAGVANSTGGAQNGDPAAKKFRPILPRQNGMPELAPKLPTLAPFVGFNPLQNPAAGKKVPPSK
KSPNAGAAAHQSGQQKLVNGGQSQPAQQKTSPPAQKNQQQVKKVSKNPTPPPPSLPAVGKMMPHPVMHSQNA
PLSIASSASAAAVASGQLDLSNFLKENLRRVHAAQAAQAAQVAAAANQSNMMYNLAQMGHMTPAMYNYQQAYF
REQLSRMQRVGNEVFNDYLQKLKTAAATGGGGPVEGELKPMLPTVTLPSPGATPPAASPKTSPLPAGKLTAAATAPQ
TKGNSSSGAANARQQTAATGNNGATVPAASLPPATKSK

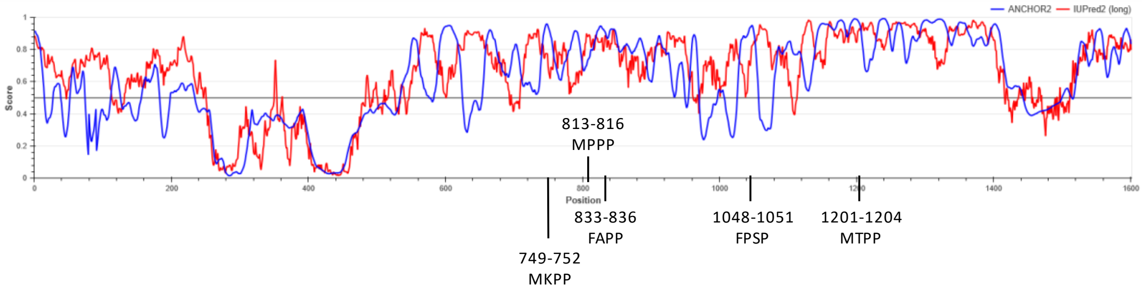

>**Prp16** (Q9VY54)
MSDDDESGVHRLEGTAGQETRGGLVIRKPKDAGAGGGGG**FKVP**QGSMLGLDKLAAKRRAEKERSERLISFKDSEFD
DTGGGSSTPQANASGASSEFA**FKKP**DTKSFEKLRGQLREHKDDTPSHTGGVSEKARERLREHIQRDRKRGPVSSTAAE
GRDRDRDWDRDRRRDRDRDRDRDRDRRQHRERERDRHRSWDRDRGRDRDRDRSMSERSVHTPREPGTPGGSSG
GISNSSWDDEDGEFGQRKSDWDMPTPRRHGNKSGDWSVRSGGSRRNHGRQDDTVRPTPAHRYNQWAHGRKRS
GATPWGEDPESLDLWEEEQRRLDREWYNIDEGYDDENNP**FGGP**NSEYFRKREEQLEQKRTKRISAQQRQNNRDNE
LWERNRMLTSGVVTLISVNDDFDEEALERVHLLVHHIIPPFLDGRIVFTKQPEPVVPVKDPTSDMALLARKGSALVRNY
REQKERRKAQKKHWELSGTKLGNIMGVQRPQDEDDMRFDKEKDKADYRKDQKFADHMRDQDTGGKSDFSRKKTI
SEQRRFLPVFASRQELLNVIRENSVIIIVGETGSGKTTQLTQYLHEDGYSKRGMIGCTQPRRVAAMSVAKRVSDEMDT
QLGEDVGYAIRFEDCTSERTVIKYMTDGILLRESLRDPELDSYSAIIMDEAHERSLSTDVLFGLLREIVARRHDLKLIVTSA
TMDSSKFATFFGNVPT**FTIP**GRTFPVDVMFSKNTCEDYVESAVKQALQVHLTPNEGDMLIFMPGQEDIEVTCEVLEE
RLAEIDNAPALSILPIYSQLPSDLQAKIFQKSSDGLRKCVVATNIAETSLTVDGIIYVIDSGYCKLKVYNPRIGMDALQIYPI
SQANANQRSGRAGRTGPGQAYRLYTQRQYKDELLALTVPEIQRTNLANTVLLLKSLGVVDLLQFHF**MDPP**PQDNILN
SLYQLWILGALDHTGALTTLGRQMAEFPLDPPQCQMLIVACRMGCSAEVLIIVSMLSVPSI**FYRP**KGREDEADGVREK
FQRPESDHLTYLNVYQQWRQNNYSSTWCNEHFIHIKAMRKVREVRQQLKDIMTQQNLSVISCGIDWDIVRKCICSA
YFYQAARLKGIGEYVNLRTGMPCHLHPTSALYGLGTTPDYVVYHELIMTAKEYMQCATAVDGYWLAELGPMFFSVK
ESGRSGREKKKQAAEHLKEMEEQMLKAQHEMEERKQQAAEREEQLATKQEIATPGNATPRRTPARIGL

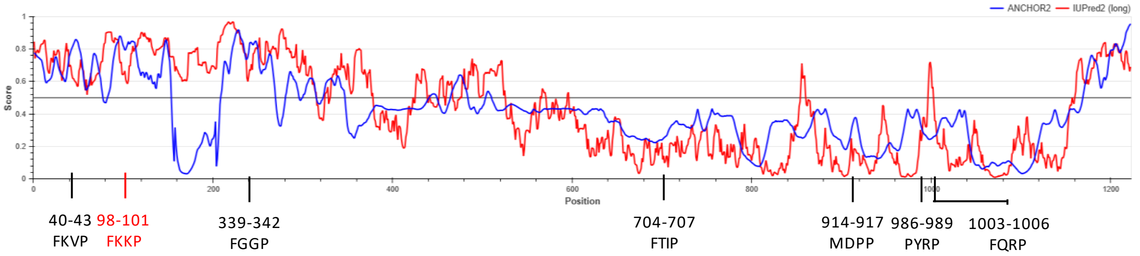

>**Sosondowah** (Q960W9)
MELPKELSVAEIRNYMLANECKVTNHALVKHFKKFLTHPQSQNEARKRFKTYVTLLSTIKNENNQKFLILRKKYVNECP
TDEVVERAVAAASGIPEPSSPGGASLN**FDSP**MRQPPPYKPPPMVTSPPAVSIAKEHQENYQECVDEFTAAIKRIEPARL
ERRTSVKSEDLDQQEKPEKTSRSNSIDVIDNKENIPRFSFSSEASSASSTSIEKPEMADPTAPAAVGDVAENPISVKEATR
KFNRMASEEEAKIISPPAKKKPEKQLIEEKDSPEVTLAHPKAKEWIVSMAKANYQELAKMASEYPELVKLQCPATGYTA
LHWAAKHGNEDVVKLIAGTYKADVNARTNGGYTPLHLATQFGRDNIFELLWNVYKANRDIMDWSGNKPLDYSRQR
SSVSASTCSKIKARKKHAIEKDLGFLRIGSLNVRVKKTTEAFSNFLGVGNGSGVAPTGYGNGGSAVAANRHHPRSHRA
PHQRHHHHVGTTRSRHPNQRAAMSTPYATNGGLPSRASVPNSRNIYDGVHKSWGSADNIPHRSEDL**MPPP**KAVEY
ISKRNKSSKRSSYASNTTDSPRDSICSSNSSSNLNGGYSSMPTTPNQLRAPKGIAASFAVDSDSDSACGFDSTWSVNCR
GSSSSNPSQS

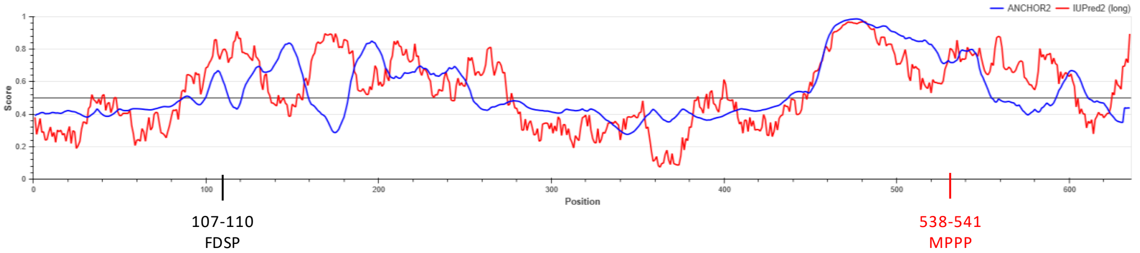

>**Stonewall** (Q9VUE5)
MSAASEVNLMLLRSVEKQTALYDRTDENYRKRLPSENAWDMVASEVGESVEKCKRRWRQLRNDYTRWCNADANR
RRNGQRRLAYPLADELRFLDRHLNIADDMAADDDRSVSSDKDRDNNRDSEGVDHHAQASLKERASSTSKLVKEVKL
ASQVRKEKSSQDKRENWENPGDKQRSRKKSAEEKLNDLEESDEPEKVPELDSFLQSDNEDDECMDEEHLEDLEGFDF
DLEQSNQEKELTPEKSLEKNNTDSTVSQPEFFVKQTRDPRLLIIGRKNSTSSFAESKASKSISGDPLETAMEDSGDEYGR
DGEKRVTGSDTTSPTRRPRRAARASISFAGIRDLPIQKPGQAQRMTRLQRRKSMSLAAVHSVRSSTSPVKMTPVPRS
QPINKPPSGPVQITKISHRDDI**FPRP**AVPSGVVNINQNQLKTQQQQKTLIFPTTDEAIANPQGSGPPQRRSVGRPPKKL
PMVQRIVAPETQTKPQSPINTKVLTIGKPTSSVASGNINNIAIKSNVVTSISSSTATTNTITKGTSNNSIKTTENVNVISYS
PNSSSTSSSSKATTGASGAVITNIPTTTTAGTSVPVGKSITQLKMTERGTQTGVQNPFSDNYFLEMIKPQMQEMNPR
QKMHFKKKVFQALMETFDDATDFPTSKELQHFNINTPSGFEHITDPELRLVRELVSMVSAAKVTLIRPPGEATAIATAS
RSGIAPEVQRGPRTNMTRQVIQRVYKPGTGQEIAPTSPAGGLDKRLFRLAAMGGKPNGNLSATQEILRKDSVDSNHS
VVKVANHAPTGSPKGAAVVAAIRPQGSLINSFFGQGNGPTTAKGAASENIRAMSRRYSVCGSSNPPNAQNASQGSA
NGSTINSNASAMEASMLKRRLIAAGHG**MVPP**TQRPRYSAVGASQMTTASQGSSLLVRKSVGCVPAYQKQISPTGGA
SLLQKTPQIASVQGSAFND**FAQP**KPAATASVNGEESSPTSALKRSLVVANTKTSFQDLLQQASQTVQRNKIESSETAAT
IAADDFSLDNLKREPVDPAEDHNDILGV

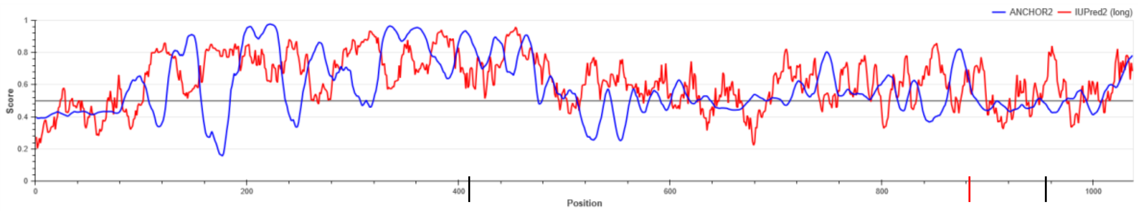

>**CG8478** (Q961C3)
MDDSRRTMAGKIDSRSSSIMTNDESVSMYLSFDAEDTLSNAKWQSALVTQNSQFLEVHNSKVEVNTDDKDLRRTILK
DLHQLTIGEPDQNSPLRPFDKSILNRHNALCSTPSKGNISTETQTPQIMESIDLTQIDDKENTQPEGCGGDNSTLSSSVD
VTANTVKANTLKTGDATMDNVSLQEAAKTPAPSQVYPLSANVVLENITEVSNEGVSMLVSPAGAEKEVAHVNEVVN
EVSELIAKALKISADSVKPATSKLKVEAGKKRQSMSSTYSGAALPRPRRSYLPTTTAETRTYSFKQRMSVVVKTTLNSPA
RKRSVGGGVSLSRRSCLPVSKLTKSSIRKSLAVTSVRSPEKIASKPAKTSTKSIPEKVFSCKNCSTTFRVKSLLDVHMRMH
DPVDNGANTLKRLNSNPVAAGVSKNRCKFCDKNFALERALHIHLMQNCDKIPPSEKRKLEFTELNHEKKAQLPKIGGT
SGINHPMTMPQKPQQRISTIPKLAPSQGTQSMAPPSVKKIPKNVAHAGVYRTPTKTVPCHICKQSFRSILEFTNHSLTV
HGNNQLKKMTGREDAQSAHD

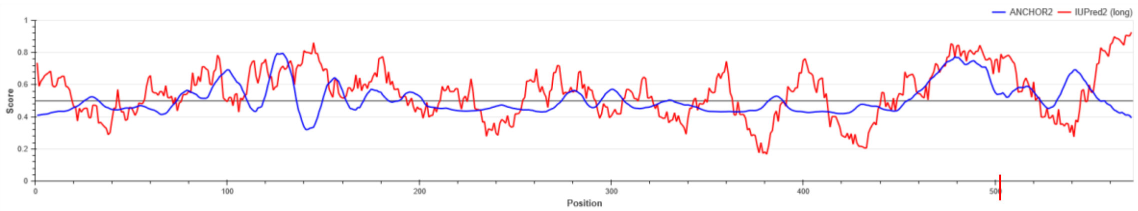

>**Centrobin** (Q9I7U5)

MSDTDTDDTDLLLLIPPNFCAEDKMSACAAADALA**MPPP**PPPATTSAAYQ**FLHP**SKKSELNSINSRLQNIALDAEEPPS
DISTISTNTVRNAGRPSRELHGMVHSTPKSGSVEPLRHRPLDDNILLEIDHYLDDNNAHRWQRHDHHLHHRSDDHLR
NERAGKEGHPGTSSLPSMRLASSSSGAVSVEAARVDRSSLNENKIISLSELWGKSSLTKTVLDNNSPNRPLCSSSLKEEQ
LRRQHLEKMVHTLQSHLLEYQQRISVAIEVDRSKDAALTEAEQTVQSLNYEVQHLRDAVHRLEADRGESQSRFDALQ
NELSQAVNLATRFQEKNDKLERELDHCRQDAKQWEERLEQLEMQLNSSKRAEELSHAELNKLRDKFAKVDYQQEKL
KARIEELEKENNTLTNQKEMLQEYHQKQKARADSLESHRKSLQETLANLTETETNLKKKLDIQQKSLKQYYQQQMEN
VVAKKMQEFQDQLDKNEEHLKNEARERERLIAERAVKQLEMINEKNNQELNLIQEKHNEEVELYRLQLANASKKIDE
MDLKLSCYKTKRADIAEKLHGVMEAQWQQALAILTTPSQNSIIQSSDTEASESPELNNARMYPETPKSSKSQRSNNTE
KNNLDVVGKRDPPSPMDKLQAYIELLLSKSPSDFDRLDEILAMTSKQGSKQSKPKSGSGNSKPPPWKC

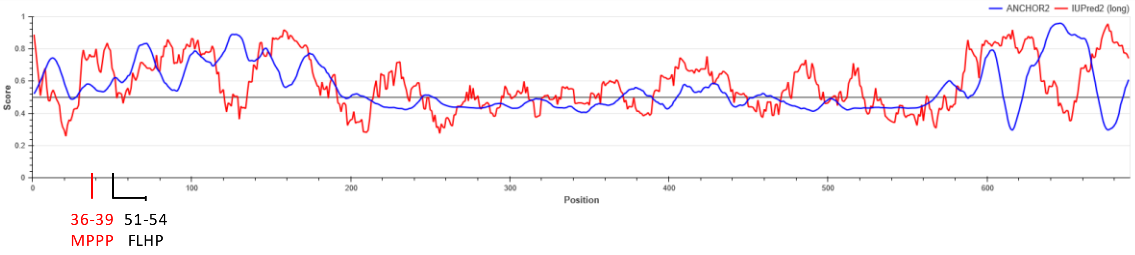

>**Miranda** (Q8IN63)

MSFSKAKLKRFNDVDVAICGSPAASNSSAGSAGSATPTASSAAAAPPTVQPERKEQIEKFFKDAVRFASSSKEAKE**FAI
P**KEDKKSKGLRL**FRTP**SLPQRLRFRPTPSHTDTATGSGSGASTAASTPLHSAATTPVKEAKSASRLKGKEALQYEIRHKN
ELIESQLSQLDVLRRHVDQLKEAEAKLREEHELATSKTDRLIEALTSENLSHKALNEQMGQEHADLLERLAAMEQQLQ
QQHDEHERQVEALVAESEALRLANELLQTANEDRQKVEEQLQAQLSALQADVAQAREHCSLEQAKTAENIELVENL
QKTNASLLADVVQLKQQIEQDALSYGQEAKSCQAELECLKVERNTLKNDLANKCTLIRSLQDELLDKNCEIDAHCDTIR
QLCREQARHTEQQQAVAKVQQQVESDLESAVEREKSYWRAELDKRQKLAENELIKIELEKQDVMVLLETTNDMLRM
RDEKLQKCEEQLRNGIDYYIQLSDALQQQLVQLKQDMAKTITEKYNYQLTLTNTRATVNILMERLKKSDADVEQYRAE
LESVQLAKGALEQSYLVLQADAEQLRQQLTESQDALNALRSSSQTLQSEERIDGDAQLAHYHELRRKDETREAYMVD
MKKALDEFATVLQFAQLELDNKEQMLVKVREECEQLKLENIALKSKQPGSASLLGTPGKANRSNTTDLEKIEDLLCDSE
LRSDCEKITTWLLNSSDKCVRQDTTSEINELLSAGKSSPRPAPRTPKAPHTPRSPRTPHTPRTPRSAASTPKKTVLFAGKE
NVPSPPQKQVLKARNI

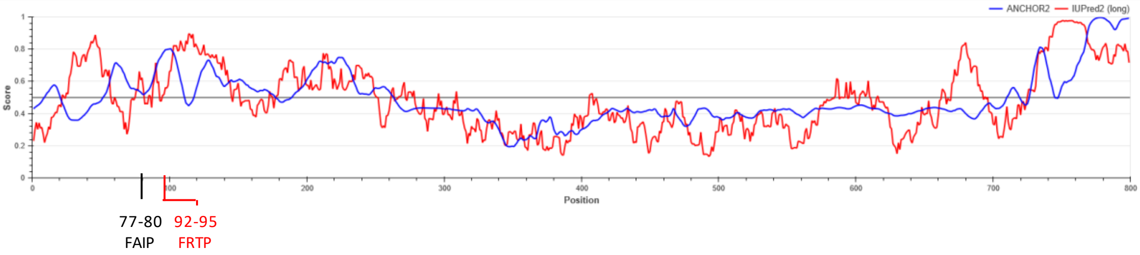

>**CENP-C** (Q9VHP9)
MSKPQNNDTLELDDILSQPVKDKERFAAFMMRKLAENKPAQNDNLFGNFKLDFDLD**FEVP**LIKKSQAKPKSKLPEVQ
PLGELVSKNSAATEKVNEPPVDQAPNENVPPRRSPTLSPNNRRSMRRSGNVPGSDKLRRHAIRRRSRSCGRQLLPEFE
ETVNLTRSISSPVNFLPEISSTPCTEKQKEEVAKNTTRVETDKPAEKPMELSQEPEPENPLQTKVTSPARNPILAAEIEQIC
KERQSSFHKNVLQLDYSGRAPYSRPPTPSSPSVAGLRRTYTMEKGPAPGQLLLSPSHRYDTPSKMPVVKAKRFNQEL
MVPDTPERQSHDPAWQSEPQPE**FVVP**ETQPQDLGELVQTLSRSAISPIVVINTSNSNRSVRRDAVAMKSVPTSPVTA
LSSPPIAPSPRRSAAASPQKSIAQLPRVEENMDAIMTDDESDEHPSTVPLNLAPSGGNTTRQRRLRSSNRARATIESQE
SSMRLLNLHKSVNAKKSKPRKTAIPLNKAPSAPINGEQFARELTRMSNYEILDLRKRNSLNEIYPLNGHRNHRSEKLILEE
EIQRELLRRNLMDEAEGLPKQQSSDDSNEDYIPVPPKTQSLRTKSNDRSQGRGRPRSTRRDLPMTTELVNYLGLSQTL
ETRRKSSKDGKRCLYTKGSSDHEDNDSLSPVKLPRLSKSIQIVPPPPVSLRYSQSLQNLPCSGKFDFDNVVMAAPPDFH
DSVNSDAIEIAPPPPEYVVNTRGRSTSGRKSNKNDLVLPPPGYEGGQEEEHDERPSQPRCTAKELQQSTQNGRRAME
NELVPPPIEYVEEENRNNEQSRRSTKNGNLVDRNTHNAVEYCEPPEPPEYDDSDHGQASILRRSGKKLQHSKQSVQK
SNKEQIIAPSYENNEDYDSDEEPIYNEEYGKEESQNKNVTRRKSDKDEMASHTLECIEGPDPNWNSSCNKQNRNHQN
ASKSKENDKLANRSSKSQKLSNPRQNAVGTEKSVALSNRGEECTEKSSDVMESLRVNTPTPPIDQNSDDVPSRNPSPS
RTLLSDDVPSTSRAALEFLQRSQNMSKSRPPDESSADVV**FKKP**LAPAPRAKSKKGKSEVDKLKLAKMPVEAEELNTTGI
RRSKRGQVPLQMSWCHTMDPSKFNFMSG**FIEP**RSKNSKTKKGNLSKAKKASATKPKPTVEKNLPDNRGPLCSSTPRI
SEKLPGAIPHSESLGLSTLTWEETEVQAEAEKVPKKRGRPKKAVGGVQTDTEAEPEPEPEPMISSVAPLTSDQEEPDVP
DEQAPYTEAALGPVV**FSTP**LRDEQEEASTKLMQWLRGVGDAPPSASMSDENASVSSANELIFCQVDGIDYAFYNTKE
KAMLGYMRFKPYQKRSMKQAKVHPLKLLVQFGEFNVETLAVGEEKEVHSVLRVGDMIEIDRGTRYSIQNAIDKVSVL
MCIRS

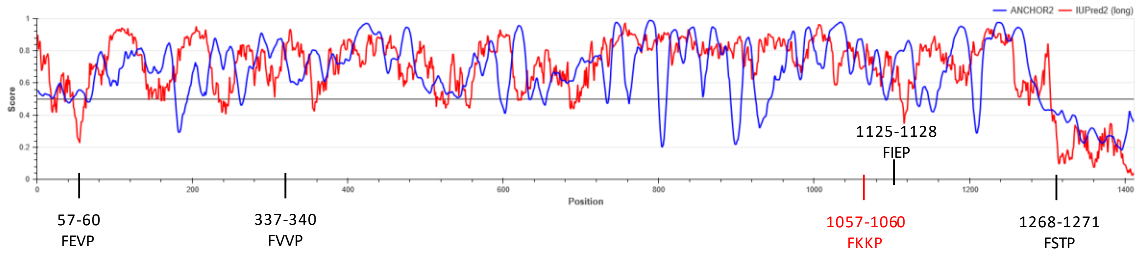

B. Smk-1-interacting proteins:

>**Zw10** (Q9W4X9)
MEEEAPRFNVLEEAFNGNGNGCANVEATQSAILKVLTRVNRFQMRVRKHIEDNYTEFMPNNTSPDIFLEESGSLNREI
HDMLENLGSEGLDALDEANVKMAGNGRQLREILLGLGVSEHVLRIDELFQCVEEAKATKDYLVLLDLVGRLRAFIYGD
DSVDGDAQVATPEVRRIFKALECYETIKVKYHVQAYMLQQSLQERFDRLVQLQCKSFPTSRCVTLQVSRDQTQLQDIV
QAL**FQEP**YNPARLCEFLLDNCIEPVIMRPVMADYSEEADGGTYVRLSLSYATKEPSSAQLRPNYKQVLENLRLLLHTLA
GINCSVSRDQHVFGIIGDHVKDKMLKLLVDECLIPAVPESTEEYQTSTLCEDVAQLEQLLVDSFIINPEQDRALGQFVEK
YETYYRNRMYRRVLETAREIIQRDLQDMVLVAPNNHSAEVANDP**FLFP**RCMISKSAQVSRSLNETIDYALMDSFSPAG
LCQTNGPHSSPAHG

>**Zwilch** (Q9VA00)
MSASANLANVYAELMRRCGESYTITYGAPPTYLVSMVGAAEAGKKIVLVFKEDRNGAVARLRTTPTRAAPKKEGSAD
LDLTGSPLKDDCLVDAIADLSIDLQLDHSNPWKLEEEYQRGIPVDKARSIVCSEFLQLAEGLGSVWFLCDGSDLGQTQL
LQYEFNPTHFSRGILSYQGVRPAYLVTSQALVRHHGKTPDETLIENSYQVNPHMRLRCSWTSSAALPLLVNLNDCDVA
LNHTFRVGDCGPLTQDFMNQLRILVYIREDIVSYHTDVKQGVSRDPTYRCGSGIDMDELRESINQTMTDVSGLIGRYSI
SNAEFDIEDVVQRAKVRQLTDLTDKLWELLKCCHSYKDLKIAFSMLFQCAARCNIVNTPTNKNRLAKIITELANRRLAM
PCLSGAEPLELLLEIGLEKLYKDYEFIYTESKMCSTNLLKDDSSGASMDDGSPQNLPQLRKSLHNAVRGDPTPGAGMRK
TLLHHHGAVNSRSTKYAGSDDDAGFKNSHFDEHESTERISKLFQIHCTLEHLLMMHIHLNLANVYNDVCSELLKKPPKL
VESIDDQLSDVMDIRLSAHYVRDHLDGKDPYSRHITMRSHNKFRELKTTFYFNSENICPPNLAQCFQCDDKEMVKERT
YHSWIYHKIRSLK

>**Spindle-A** (Q27297)
MEKLTNVQAQQEEEEEEGPLSVTKLIGGSITAKDIKLLQQASLHTVESVANATKKQLMAIPGLGGGKVEQIITEANKLV
PLGFLSARTFYQMRADVVQLSTGSKELDKLLGGGIETGSITEIFGEFRCGKTQLCHTLAVTCQLPISQKGGEGKCMYIDT
ENTFRPERLAAIAQRYKLNESEVLDNVAFTRAHNSDQQTKLIQMAAGMLFESRYALLIVDSAMALYRSDYIGRGELAA
RQNHLGLFLRMLQRLADEFGVAVVITNQVTASLDGAPGMFDAKKPIGGHIMAHSSTTRLYLRKGKGETRICKIYDSPC
LPESEAMFAILPDGIGDARES

>**RfC4** (P53034)
MPEEPEKTADDKRSHLPWIEKYRPVKFKEIVGNEDTVARLSVFATQGNAPNIIIAGPPGVGKTTTIQCLARILLGDSYKE
AVLELNASNERGIDVVRNKIKMFAQQKVTLPRGRHKIVILDEADSMTEGAQQALRRTMEIYSSTTRFALACNTSEKIIE
PIQSRCAMLRFTKLSDAQVLAKLIEVAKWEKLNYTEDGLEAIVFTAQGDMRQGLNNLQSTAQGFGDITAENVFKVCD
EPHPKLLEEMIHHCAANDIHKAYKILAKLWKLGYSPEDIIANIFRVCKRINIDEHLKLDFIREIGITHMKIIDGINSLLQLTAL
LAKLCIAAEKH

>**Nipsnap** (Q9VXK0)
LRQLYCILFCFLLTLTFHLNIILVCNTPANNYKILHKSKMLKLRNLLAVGKSNNNAVRSLSTTPSRNDSESWFSKLLVRKIE
PTKESHSRMLSDKEIIYALHTHNVRPDSMGSYLNNYKTTVALINEKKANLSCELVASWTVQVGDMDQCLHLWKYTG
GFEKIDQAKEDLWNDPEYLSLMQERSKFLRSRHLQYLLAFSYWPQIASRTGKNIYEMRSYRLTPGTMIEWGNNWAR
AINYRKHNNEAFAGFFSQIGRLYNVHHIWCYKSLQDRKETREAAWRSPGWDECVAYTVPLIREMHCRVLAPTEFSPS
Q

>**Licorne** (O62602)
MSKRHRLTPFTIAKEPEAAIVPPRNLDSRATIQIGDRTFDIDADSLEKICDLGRGAYGIVDKMRHKQTDTVLAVKRIPM
TVNIREQHRLVMDLDISMRSSDCPYTVHFYGAMYREGDVWICMEVMSTSLDKFYPKVFLHDLRMEESVLGKIAMSV
VSALHYLHAQLKVIHRDVKPSNILINRAGQVKICDFGISGYLVDSIAKTIDAGCKPYMAPERIDPQGNPAQYDIRSDVW
SLGIGMIEMATGRYPYDNWRTPFEQLRQVVEDSPPRLPEGTFSPEFEDFIAVCLQKEYMARPNYEQLLKHSFIVEHLQ
RNTDISEFVARILDLPDAQPAQ

>**Grip75** (Q9VKU7)
MIHDLLLACRSHNPEQLGIKAFNETTVIDQFIHPCEREIFMDIIKIIKVYQEVEQFTHSSGRKSDTHGELPDSLHGYYLLNL
AKGIEMALEEYYAEIGRLEKYCLGNERNSLSYVYNALYAKFPLLVFMRNLITEIHVLNLRGCVLLHNLHQQCEHGDIQLE
KAIKIIMKPVKNAFFSSLAHWLLFGVIDDVHSEFFIKFTPTDAVDGSSFSKSATCSLLSAEKNPEDYIWQYEVNMSQLPG
FFSIVLAEKVLFVGQTVLVFKMGRNVKVKNKTDPLAAKLAELDSDDIYQLWSGRESEFFKMVVDLSNEDTINVFRLEKV
IIDIKNYVSARLSEIAVNEVDLERQMGLIKDFFLLGRGEFYLEFCSQMVGTMETYREERFKNVTRSFELAATVTGITDDL
DKFSLICQRSTSEPDDTSDFNFLQGLSLKYEYEWPLNLLFSPTTIERYNNIFRFLLIIRTYQYEIQRVWAKQTWRAKSAKD
VPPNNKIITLRNYLMFFLNNMQYYIQVDVLESQFGILMNVIKSRSDFEVIQRAHTVFLANVLSHCFLLNESETQLNVTG
SQNRNPIYGTLLKLFGICEKFAHMTQTKDPSDDLEDEVDQLNESFGVQIASLIQLLVDVKSASCLGPLSQLLLRLDFNC
WFSASHNTSA

>**γTub23C** (P23257)
MPSEIITLQLGQCGNQIGFEFWKRLCLEHGISPSGVLEDFANDGLDRKDVFFYQADDDHYIPRAVLLDLEPRVINTIMG
SVYSKLYNPENVYLSKHGGGAGNNWASGYSQGEKLQEEVFDIIDREADGSDSLEGFILCHSIAGGTGSGMGSFIMERL
ADRYPKKLIQTFSVFPNQDEISDVVVQPYNSMLTLKRLTTAADSVVVLDNTALNRIACDRLHIQNPSFSQINNLVSTIM
SVSTTTLRYPSYMNNNLIGLTAPLIPTPQLHFLMTGYTPLTSDSDIHTQQLVNVRKTTVLDVMRRLLQPKNMMVSTGP
DKSNHHCYISILNIIQGEVDPTQVHKSLQRIRDRKMAQFIPWGPTSIQVALSRSSPYVQSNHRVSGLMLANHTSICSLF
ERALNQYDKLRKRGAFLDQFRREDIFKDDLNELDESRETVDCLVQEYEAATREDYMQFSVKRGNGPVDSKSEDSRSVT
SAGS

Figure S3. Protein sequences of EVH1 and Smk-1 interacting proteins. A. Collection of protein sequences of the EVH1-interacting proteins Incenp, Psc, Prp16, Sowah, Stwl, CG8478, Centrobin, Miranda and CENP-C (with their Uniprot identifiers in parenthesis). All FxxP or MxPP sequences are underlined and highlighted. The identified EVH1-binding SLiMs are indicated in bold red. Intrinsically disordered regions (above the grey line, score >0.5) of the proteins are predicted using the IUPred2A combined web interface (diagrams are shown under the sequences of the proteins): IUPred2A curve (red) represents the energy estimation based predictions for ordered and disordered residues, while the ANCHOR2 curve (blue) represents the disordered binding regions. The exact localisation of the FxxP or MxPP sequences are indicated below the diagram. The identified SLiMs (in red) lay in disordered regions of the proteins. **B**. A collection of protein sequences of the Smk-1-interacting proteins Zw10, Zwilch, Spindle-A, Rfc4, Nipsnap, Licorne, Grip75 and γTub23C (with their Uniprot identifiers in parenthesis). FxxP motifs are found only in Zw10 (underlined and highlighted).

Figure S4

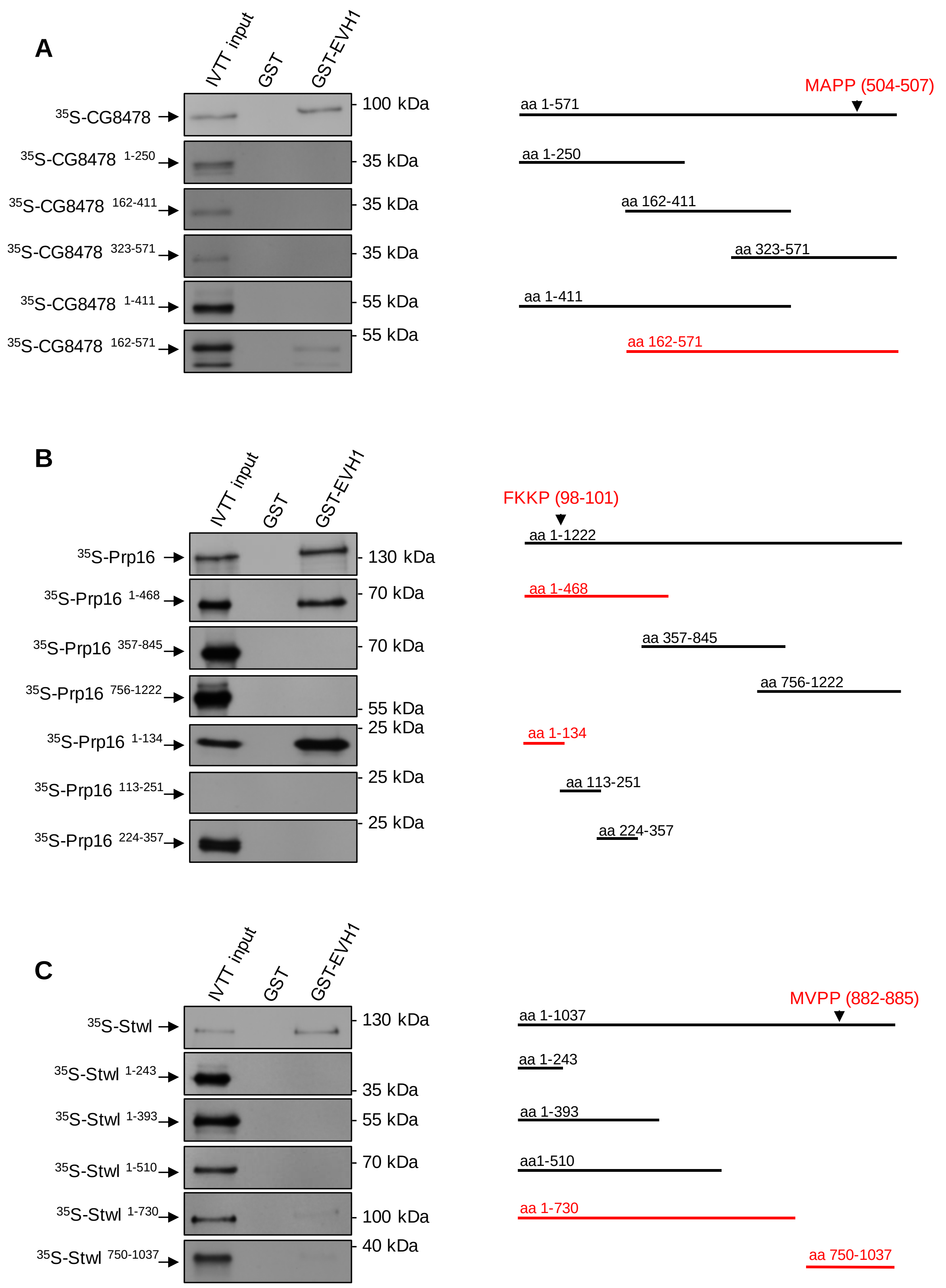
A
IVTT input
GST
GST-EVH1
35S-CG8478
35S-CG8478 1-250
35S-CG8478 162-411
35S-CG8478 323-571
35S-CG8478 1-411
35S-CG8478 162-571
100 kDa
35 kDa
35 kDa
35 kDa
55 kDa
55 kDa
MAPP (504-507)
aa 1-571
aa 1-250
aa 162-411
aa 323-571
aa 1-411
aa 162-571
B
IVTT input
GST
GST-EVH1
35S-Prp16
35S-Prp16 1-468
35S-Prp16 357-845
35S-Prp16 756-1222
35S-Prp16 1-134
35S-Prp16 113-251
35S-Prp16 224-357
130 kDa
70 kDa
70 kDa
55 kDa
25 kDa
25 kDa
25 kDa
FKKP (98-101)
aa 1-1222
aa 1-468
aa 357-845
aa 756-1222
aa 1-134
aa 113-251
aa 224-357
C
IVTT input
GST
GST-EVH1
35S-Stwl
35S-Stwl 1-243
35S-Stwl 1-393
35S-Stwl 1-510
35S-Stwl 1-730
35S-Stwl 750-1037
130 kDa
35 kDa
55 kDa
70 kDa
100 kDa
40 kDa
MVPP (882-885)
aa 1-1037
aa 1-243
aa 1-393
aa1-510
aa 1-730
aa 750-1037

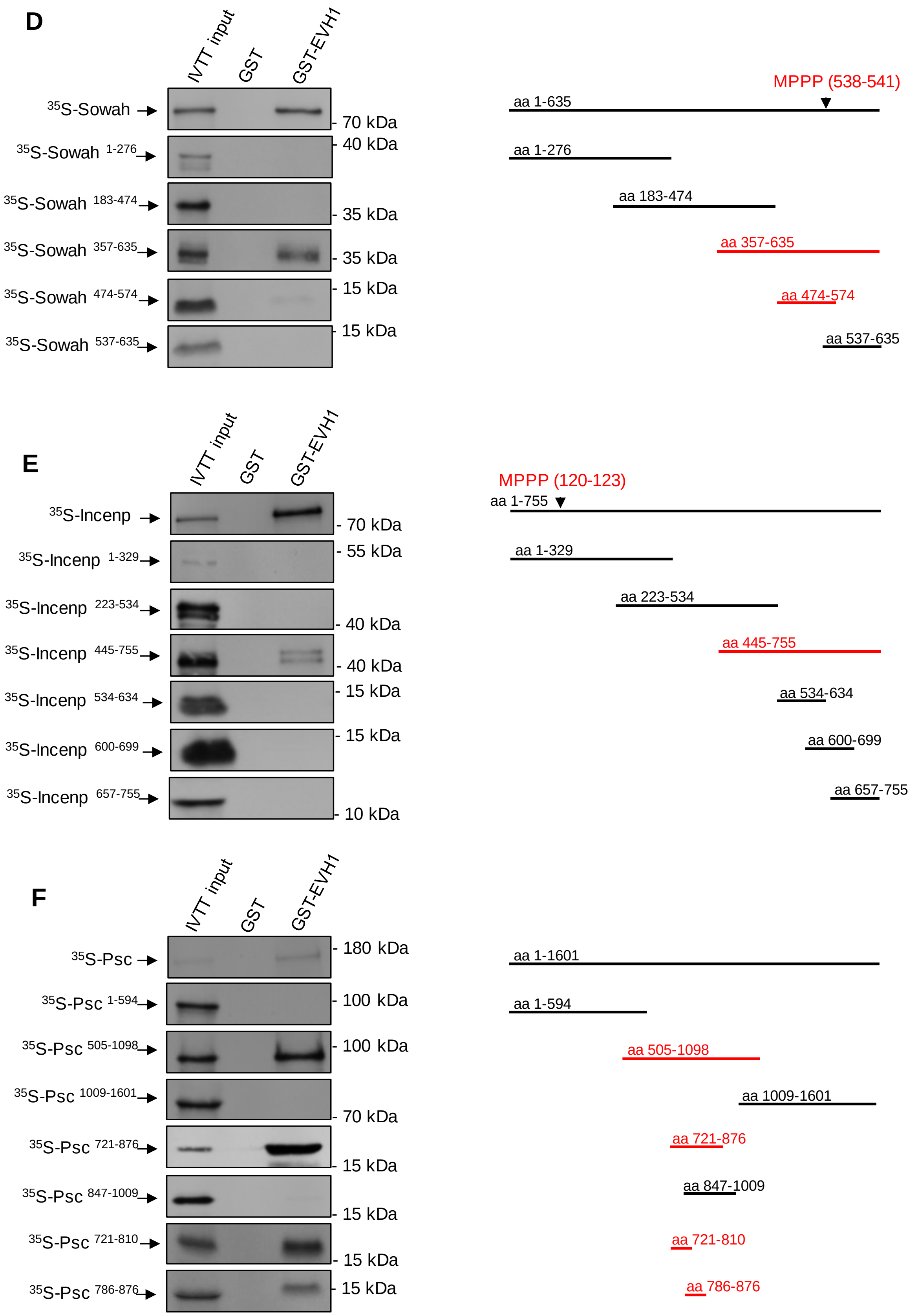
D
IVTT input
GST
GST-EVH1
35S-Sowah
35S-Sowah 1-276
35S-Sowah 183-474
35S-Sowah 357-635
35S-Sowah 474-574
35S-Sowah 537-635
70 kDa
40 kDa
35 kDa
35 kDa
15 kDa
15 kDa
MPPP (538-541)
aa 1-635
aa 1-276
aa 183-474
aa 357-635
aa 474-574
aa 537-635
E
IVTT input
GST
GST-EVH1
35S-Incenp
35S-Incenp 1-329
35S-Incenp 223-534
35S-Incenp 445-755
35S-Incenp 534-634
35S-Incenp 600-699
35S-Incenp 657-755
70 kDa
55 kDa
40 kDa
40 kDa
15 kDa
15 kDa
10 kDa
MPPP (120-123)
aa 1-755
aa 1-329
aa 223-534
aa 445-755
aa 534-634
aa 600-699
aa 657-755
F
IVTT input
GST
GST-EVH1
35S-Psc
35S-Psc 1-594
35S-Psc 505-1098
35S-Psc 1009-1601
35S-Psc 721-876
35S-Psc 847-1009
35S-Psc 721-810
35S-Psc 786-876
180 kDa
100 kDa
100 kDa
70 kDa
15 kDa
15 kDa
15 kDa
15 kDa
aa 1-1601
aa 1-594
aa 505-1098
aa 1009-1601
aa 721-876
aa 847-1009
aa 721-810
aa 786-876

Figure S4. Mapping the EVH1-interacting regions of target proteins. A-F. *In vitro* binding of GST-EVH1 and ^{35}S-methionine-labelled overlapping pieces of the indicated proteins were analysed by SDS-PAGE followed by autoradiography. GST served as negative control. Interacting fragments (and EVH1-binding SLiMs) are indicated in red. The MPPP (aa 120-123) SLiM in Incenp lies outside of the mapped region (aa 445-755), which bound weakly to the EVH1 domain (**E**). In the case of Stwl both aa 1-730 and aa 750-1037 (containing the MVPP (aa 882-885) SLiM) fragments bind to the bait (**C**). Within the EVH1-binding region of Psc (aa 721-876) no PP4-SLiMs were identified (**F**).

Figure S5

A

```
CLUSTAL O(1.2.4) multiple sequence alignment


Dm_Centrobin      MSDTDTDDTDLLLLIPPNFCAEDKMSACAAADALAMPPPPPPA------TTSAAYQFLHP       54
Hs_Centrobin      MA----------------TSADSPSSPLGAEDLLSDSSEPPGLNQVSSEVTSQLYASLRL       44
                  *:                .*:.  *  .* * *:    **        .**  *  *:

Dm_Centrobin      SKKSELNSINSRLQNIALDAEEPPSDISTISTNTVRNAGRPSRELHGMVHSTPKSGSVEP       114
Hs_Centrobin      SRQAEAT---AR-AQLYLP-----------------STSPPHEGLDGFAQELSRSLS---       80
                  *:::* .   :*  :: *                  .:. * . *.*:.:.  :* *

Dm_Centrobin      LRHRPLDDNILLEIDHYLD--DNNAHRWQRHD------HHLHHRSDDHLRNERAGKEGHP       166
Hs_Centrobin      -----------VGLEKNLKKKDGSKHIFEMESVRGQLQTMLQTSRDTAYRDP-----LIP       124
                             : ::: *.  *.. * :: ..        *:   *   *:        *

Dm_Centrobin      GTSSLPS--MRLASSSSGAVSVEAARVDRSSLNENKIISLSELWGKSSLTKTVLDNNSPN       224
Hs_Centrobin      GAGSERREEDSFDSDSTA-TLLNTRPLQ-DLSPSSSAQALEELFPRYTSLRPGPPLNPPD       182
                  *:.*         : *.*:. . :::  :: .   ...  :*.**: : :  :     * *:

Dm_Centrobin      RPLCSSSLKEEQLRRQHLEKMVHTLQSHLLEYQQRISVAIEVDRSKDAALTEAEQTVQSL       284
Hs_Centrobin      FQGLRDALDSEHTRRKHCERHIQSLQTRVLELQQQLAVAVAADRKKDTMIEQLDKTLARV       242
                       .:*..*: **:* *: :::**:::** **:::**: .**.**: : : ::*:  :

Dm_Centrobin      NYEVQHLRDAVHRLEADRG------------------ESQSRFDALQNELSQAVNLATRF       326
Hs_Centrobin      VE-------GWNRHEAERTEVLRGLQEEHQAAELTRSKQQETVTRLEQSLSEAMEALNRE       295
                         . :* **:*                   :.*. .   *::.**:*::  .*

Dm_Centrobin      QEKNDKLERELDHCRQDAKQWEERLEQLEMQLNSS-------------KRA-EELSHAEL       372
Hs_Centrobin      QESARLQQRERE-------TLEEERQALTLRLEAEQQRCCVLQEERDAARAGQLSEHREL       348
                  **.    :** :          **. : * ::*::.              ** :  .* **

Dm_Centrobin      NKLRDKFAKV--DY--QQEKLKARIEELEKENNT-LTNQ-----KEMLQEYHQKQKARAD       422
Hs_Centrobin      ETLRAALEEERQTWAQQEHQLKEHYQALQEESQAQLEREKEKSQREAQAAWETQHQ--LA       406
                  :.**  : :    :  *:.:** : : *::*.:: * .:      :*    :. :::

Dm_Centrobin      SLESHRKSLQETLANLTETETNLKKKLDIQQKSLKQYYQQ-----------QMENVVAKK       471
Hs_Centrobin      LVQSEVRRLEGELDTARR----ERDALQLEMSLVQARYESQRIQLESELAVQLEQRVTER       462
                   ::*. : *:  * .  .      :. *::: . ::  *:.            *:*: *:::

Dm_Centrobin      MQEFQDQLDKNEEHLKNEARERERLIAERAVKQLEMINEKNNQEL-----------NLIQ       520
Hs_Centrobin      LAQAQE------SSLRQAA-----SLREHHRKQLQDLSGQHQQELASQLAQFKVEMAERE       511
                  : : *:       . *:: *       : *:  ***: :. :::***              :

Dm_Centrobin      EKHNEEVELYRLQLANASKKIDEMDLKLSCYKTKRADIAEKLHGVMEAQWQQALAILTTP       580
Hs_Centrobin      ERQQQVAEDYELRLAREQARVCELQSGNQQLEEQRVELVERLQAMLQAHWDEANQLLSTT       571
                  *:::: .* *.*:**. . :: *::   .  : :*.::.*:*:.:::*:*::*  :*:*

Dm_Centrobin      SQNSII----QSSDTEASESPELNNARMYPETPKSSKSQRSNNTE---------------       621
Hs_Centrobin      LPPPNPPAPPAGPSSPGPQEPEKEERRVWTMPPMAVALKPVLQQSREARDELPGAPPVLC       631
                              . .: . :.** :: *::   * :   :   : .

Dm_Centrobin      -----------------------------------------------------------K       622
Hs_Centrobin      SSSSDLSLLLGPSFQSQHSFQPLEPKPDLTSSTAGAFSALGAFHPDHRAERPFPEEDPGP       691


Dm_Centrobin      NNLDVVGKRDPPSPMDKLQAYIELLLSKSPSDFDRLDEILAMTSKQGSKQSKPKSGSGNS       682
Hs_Centrobin      DGEGLLKQGLPPAQLEGLKNFLHQLLETVPQNNENPS----------VDLL--PPKSGPL       739
                  :. .:: :  **: :: *: ::. **.. *.: :. .              .       **

Dm_Centrobin      KPPPWKC-----------------------------------------------------       689
Hs_Centrobin      TVPSWEEAPQVPRIPPPVHKTKVPLAMASSLFRVPEPPSSHSQGSGPSSGSPERGGDGLT       799
                  . * *:

Dm_Centrobin      ------------------------------------------------------------       689
Hs_Centrobin      FPRQLMEVSQLLRLYQARGWGALPAEDLLLYLKRLEHSGTDGRGDNVPRRNTDSRLGEIP       859


Dm_Centrobin      --------------------------------------------       689
Hs_Centrobin      RKEIPSQAVPRRLATAPKTEKPPARKKSGHPAPSSMRSRGGVWR       903
```

B

```
CLUSTAL O(1.2.4) multiple sequence alignment


Dm_Prp16      -MSDDDESGVHRLEGTAGQETRGGLVIRKPKDAGAGGGGGFKVPQGSMLGLDKLAAKRRA   59
Hs_Prp16      MGDTSEDASIHRLEGTDLDCQVGGLICKSKSAASEQHVFKAPAPRPSLLGLDLLASLKRR   60
                . .:::.:******  :   ***: :. . *.        .*: *:**** **: :*

Dm_Prp16      EKERSE--------RLISFKDSEFDDTGGGSSTPQANASGASSEFAFKKPDTKSFEKLRG   111
Hs_Prp16      EREEKDDGEDKKKSKVSSYKDWEESKDDQ--KDA-EEEGGDQAG---------QNIRKDR   108
              *:*..:        :: *:** * .. .    .    : .* .:          .  :

Dm_Prp16      QLREHKDDTPSHTGGVSEKARERLREHIQRDRKRGPVSSTAAEGRDRDRDWDRDRRRDRD   171
Hs_Prp16      HYRSARVETPSHPGGVSEEFWERSRQREREREHGVYA-----SSKEEKDWKKEKSRDRD   163
              : *. : :**** *****:  ** *:: :. *::*  :      . ..::**.::: ****

Dm_Prp16      RDRDRDRDRRQHREREDRHRS-WDRDRGRDRDRDRSMSERSVHTPREPGTPGGSSGGIS   230
Hs_Prp16      YDRKRDRDERDR-----SRHSSRSERDGGSERSSRRNEPESPRHRPKDA-------ATPS   211
               **.****.*::     .** *  :** * :*.  *.  *   * *::        .  *

Dm_Prp16      NSSWDDEDGEFGQ-RKSDWDMPTPRRHGNKS---GDWSVRSGGSRRNHGRQDDTVRPTPA   286
Hs_Prp16      RSTWEEEDSGYGSSRRSQWESPSPTPSYRDSERSHRLSTRDRDRSVRGKYSDDTPLPTPS   271
              .*:*::**. :*. *:*:*: *:*    ..*       *.*. .    .   .***  ***:

Dm_Prp16      HRYNQWAHGRKRSGATPWGE----------------DPESLDLWEEEQRRLDREWYNIDE   330
Hs_Prp16      YKYNEWADDRRHLGSTPRLSRGRGRREEGEEGISFDTEEERQQWEDDQRQADRDWYMMDE   331
              ::**:**..*:: *:**  .                 *. : **::**: **:** :**

Dm_Prp16      GYDDENNPFGGPNSEYFRKREEQLEQKRTKRISAQQRQNNRDNELWERNRMLTSGVVTLI   390
Hs_Prp16      GYDEFHNPLAYSSEDYVRRREQHLHKQKQKRISAQRRQINEDNERWETNRMLTSGVVHRL   391
              ***: :**:.  ..:*.*:**::*.::: ******:** *.*** ** *********  :

Dm_Prp16      SVNDDFDEEALERVHLLVHHIIPPFLDGRIVFTKQPEPVVPVKDPTSDMALLARKGSALV   450
Hs_Prp16      EVDEDFEEDNAAKVHLMVHNLVPPFLDGRIVFTKQPEPVIPVKDATSDLAIIARKGSQTV   451
              .*::**:*:    :***:**:::*****************:**** ***:*::*****  *

Dm_Prp16      RNYREQKERRKAQKKHWELSGTKLGNIMGVQRPQDEDDMRFDKEKDKADYRKDQKFADHM   510
Hs_Prp16      RKHREQKERKKAQHKHWELAGTKLGDIMGVKKEEEPDKA--VTEDGKVDYRTEQKFADHM   509
              *::******:***:*****:*****:****:: :: *.     .*..*.***.:*******

Dm_Prp16      RDQDTGGKSDFSRKKTISEQRRFLPVFASRQELLNVIRENSVIIIVGETGSGKTTQLTQY   570
Hs_Prp16      KRKS-EASSEFAKKKSILEQRQYLPIFAVQQELLTIIRDNSIVIVVGETGSGKTTQLTQY   568
              : :.   ..*:*::**:* ***::**:** :****.:**:**::*:**************

Dm_Prp16      LHEDGYSKRGMIGCTQPRRVAAMSVAKRVSDEMDTQLGEDVGYAIRFEDCTSERTVIKYM   630
Hs_Prp16      LHEDGYTDYGMIGCTQPRRVAAMSVAKRVSEEMGGNLGEEVGYAIRFEDCTSEN-TIKYM   627
              ******:. ********************:**. :***:*************. .****

Dm_Prp16      TDGILLRESLRDPELDSYSAIIMDEAHERSLSTDVLFGLLREIVARRHDLKLIVTSATMD   690
Hs_Prp16      TDGILLRESLREADLDHYSAIIMDEAHERSLNTDVLFGLLREVVARRSDLKLIVTSATMD   687
              ***********: :** **************.**********:**** ************

Dm_Prp16      SSKFATFFGNVPTFTIPGRTFPVDVMFSKNTCEDYVESAVKQALQVHLTPNEGDMLIFMP   750
Hs_Prp16      AEKFAAFFGNVPIFHIPGRTFPVDILFSKTPQEDYVEAAVKQSLQVHLSGAPGDILIFMP   747
              :.***:****** * *********::***.   *****:****:*****:    **:*****

Dm_Prp16      GQEDIEVTCEVLEERLAEIDNAPALSILPIYSQLPSDLQAKIFQKSSDGLRKCVVATNIA   810
Hs_Prp16      GQEDIEVTSDQIVEHLEELENAPALAVLPIYSQLPSDLQAKIFQKAPDGVRKCIVATNIA   807
              ********.: : *:* *::*****::*****************: **:***:******

Dm_Prp16      ETSLTVDGIIYVIDSGYCKLKVYNPRIGMDALQIYPISQANANQRSGRAGRTGPGQAYRL   870
Hs_Prp16      ETSLTVDGIMFVIDSGYCKLKVFNPRIGMDALQIYPISQANANQRSGRAGRTGPGQCFRL   867
              *********::***********:*********************************.:**

Dm_Prp16      YTQRQYKDELLALTVPEIQRTNLANTVLLLKSLGVVDLLQFHFMDPPPQDNILNSLYQLW   930
Hs_Prp16      YTQSAYKNELLTTTVPEIQRTNLANVVLLLKSLGVQDLLQFHFMDPPPEDNMLNSMYQLW   927
              ***  **:***: ************.********* ************:**:***:****

Dm_Prp16      ILGALDHTGALTTLGRQMAEFPLDPPQCQMLIVACRMGCSAEVLIIVSMLSVPSIFYRPK   990
Hs_Prp16      ILGALDNTGGLTSTGRLMVEFPLDPALSKMLIVSCDMGCSSEILLIVSMLSVPAIFYRPK   987
              ******:**.**: ** *.******  .:****:* ****:*:*:*********:******

Dm_Prp16      GREDEADGVREKFQRPESDHLTYLNVYQQWRQNNYSSTWCNEHFIHIKAMRKVREVRQQL   1050
Hs_Prp16      GREEESDQIREKFAVPESDHLTYLNVYLQWKNNNYSTIWCNDHFIHAKAMRKVREVRAQL   1047
              ***:*:* :****  ************ **::****: ***:**** ********** **

Dm_Prp16      KDIMTQQNLSVISCGIDWDIVRKCICSAYFYQAARLKGIGEYVNLRTGMPCHLHPTSALY   1110
Hs_Prp16      KDIMVQQRMSLASCGTDWDIVRKCICAAYFHQAAKLKGIGEYVNIRTGMPCHLHPTSSLF   1107
              ****.**.:*: *** **********:***:***:*********:************:*:

Dm_Prp16      GLGTTPDYVVYHELIMTAKEYMQCATAVDGYWLAELGPMFFSVKESGRSGREKKKQAAEH   1170
Hs_Prp16      GMGYTPDYIVYHELVMTTKEYMQCVTAVDGEWLAELGPMFYSVKQAGKSRQENRRRAKEE   1167
              *:* ****:*****:**:******.***** *********:***::*:* :*::::* *.

Dm_Prp16      LKEMEEQMLKAQHEMEERKQQAAERE-------EQLATKQEIATPGNATPRRTPARIGL   1222
Hs_Prp16      ASAMEEEMALAEEQLRARRQEQEKRSPLGSVRSTKIYTPGRKEQGEPMTPRRTPARFGL   1226
               . ***:*  *:.::. *:*:  :*.         :: *  .       *******:**
```

C

```
                          10/12/11  17/20/19
S.cer[25-140]     --NTEPKRVKVYIL-ENNEWKDTGTGFCIGEVDEGKFAYLVVSDEDSPTETLLKSKLEGN     81
D.mel[1-123]      MTTDTRRRVKLYALNAERQWDDRGTGHVSSTYVERLKGISLLVRAESDGSLLLESKIQPD     60
H.sap[1-122]      -MTDTRRRVKVYTLNEDRQWDDRGTGHVSSGYVERLKGMSLLVRAESDGSLLLESKINPN     59

                        91/70/69
S.cer[25-140]     IEYQRQEETLIVWKDLGGKDIALSFEESMGCDTLCEFIVHVQRNIESN-ISLVTVKSSDN     140
D.mel[1-123]      TAYQKQQDTLIVWSEGDNFDLALSFQEKAGCDEIWEKICQVQGKDPSVEITQDIVEESED     120
H.sap[1-122]      TAYQKQQDTLIVWSEAENYDLALSFQEKAGCDEIWEKICQVQGKDPSVDITQDLVDESEE     119

S.cer[25-140]     ---       140
D.mel[1-123]      ERF       123
H.sap[1-122]      ERF       122
```

Figure S5. Multiple sequence alignments. A. Sequence alignment of the fruit fly (Uniprot: Q9I7U5) and human (Uniprot: Q8N137) Centrobin proteins. The MPPP (in fruit flies) and FRVP (in humans) SLiMs are highlighted in green. **B.** Sequence alignment of the fruit fly (Uniprot: Q9VY54) and human (Uniprot: Q92620) Prp16 proteins. The FKKP (in fruit flies) and FKAP (in humans) SLiMs are highlighted in green. The ATP-dependent RNA helicase domain shows high level conservation and visualised in red. Symbols: * (asterisk) indicates conserved residues; : (colon) indicates strongly similar residues; . (period) indicates weakly similar residues. **C.** Sequence alignment of the EVH1 domains of yeast Psy2 (Uniprot: P40164), fruit fly Falafel (Uniprot: Q9VFS5) and human SMEK1 (Uniprot: Q6IN85). The conserved Tyr (10/12/11 yeast/fruit fly/human numbering) and Trp (17/20/19 yeast/fruit fly/human numbering) residues are highlighted in green and the invariant Leu (91/70/69 yeast/fruit fly/human numbering) are highlighted in yellow.

Figure S6

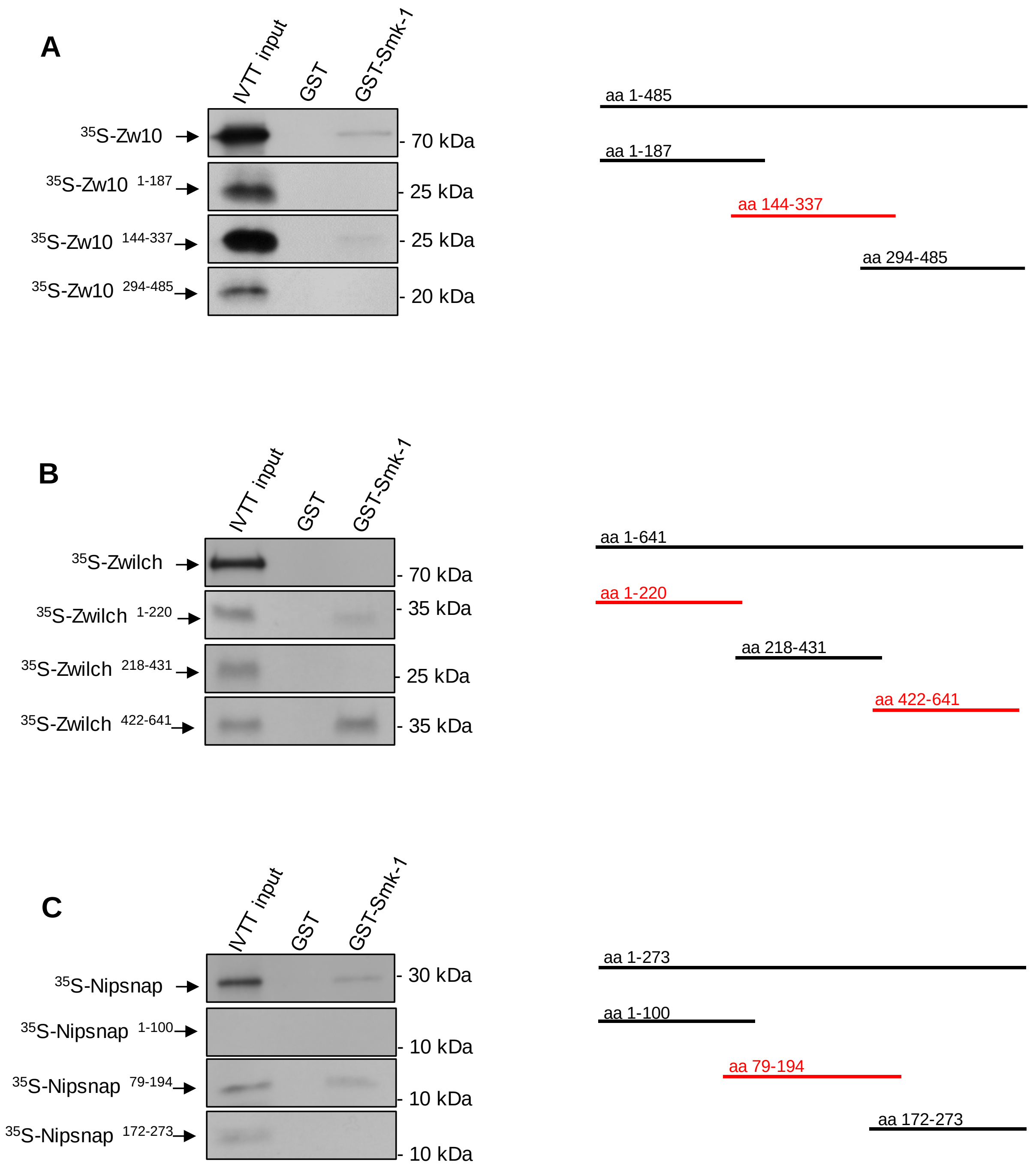
A
IVTT input
GST
GST-Smk-1
35S-Zw10
35S-Zw10 1-187
35S-Zw10 144-337
35S-Zw10 294-485
70 kDa
25 kDa
25 kDa
20 kDa
aa 1-485
aa 1-187
aa 144-337
aa 294-485
B
IVTT input
GST
GST-Smk-1
35S-Zwilch
35S-Zwilch 1-220
35S-Zwilch 218-431
35S-Zwilch 422-641
70 kDa
35 kDa
25 kDa
35 kDa
aa 1-641
aa 1-220
aa 218-431
aa 422-641
C
IVTT input
GST
GST-Smk-1
35S-Nipsnap
35S-Nipsnap 1-100
35S-Nipsnap 79-194
35S-Nipsnap 172-273
30 kDa
10 kDa
10 kDa
10 kDa
aa 1-273
aa 1-100
aa 79-194
aa 172-273

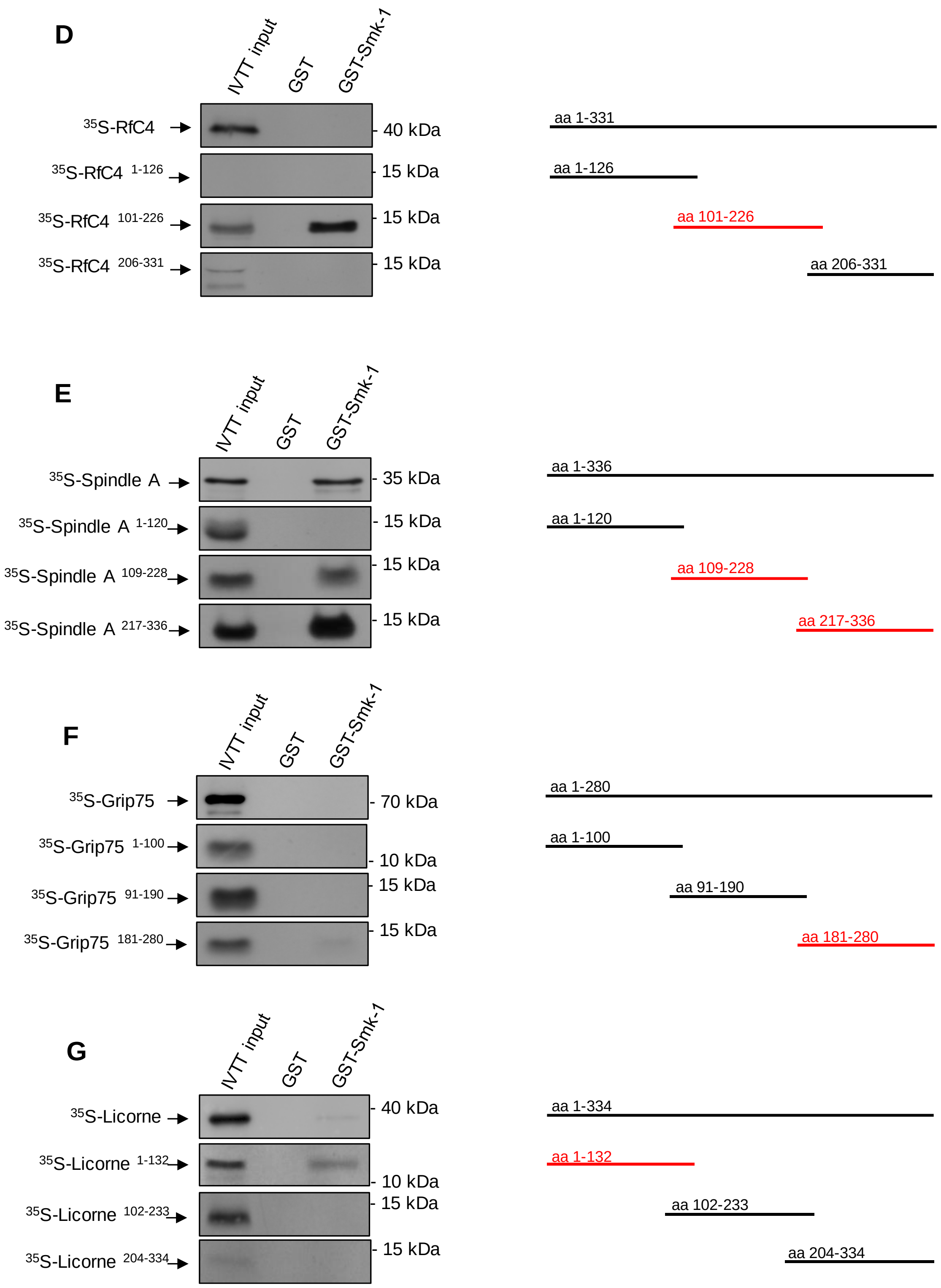

Figure S6. Mapping the Smk-1-interacting regions of target proteins. A-G. *In vitro* binding of GST-Smk-1 and ^{35}S-methionine-labelled overlapping pieces of the indicated proteins were analysed by SDS-PAGE followed by autoradiography. GST served as negative control. Interacting fragments are shown in red.

TABLES

Table S1

		AP-MS								
		GST CTRL Embryo		GST-EVH1 Embryo		GST-Smk-1 Embryo		*In vitro* Protein-Protein Interaction		
**CG number**	**Name**	**Score**	**# pep**	**Score**	**# pep**	**Score**	**# pep**	**Selected for *in vitro* testing**	**Interaction with EVH1**	**Interaction with Smk-1**
CG9351	Falafel (bait)	-	-	20654	348	31544	444	-		
CG2890	PP4R2	-	-	-	-	-	-	-		
CG32505	PP4c	-	-	-	-	-	-	-		
CG9484	Hyperplastic discs	-	-	1804	33	184	3	-		
CG32604	Prp16	-	-	1540	29	-	-	+	+	
CG12249	Miranda	-	-	1021	17	-	-	+	+	
CG8478	CG8478	-	-	1001	16	161	2	+	+	
CG3886	Posterior sex combs (Psc)	-	-	942	17	-	-	+	+	
CG5581	Otefin	-	-	770	13	142	1	+	-	
CG5753	Staufen	-	-	396	5	-	-	+	-	
CG31258	Cenp-C	-	-	396	8	-	-	+	+	
CG6620	Aurora B	-	-	367	6	-	-	-		
CG4454	Borealin-related	-	-	337	7	-	-	+	-	
CG11207	Fascetto	-	-	313	6	-	-	+	-	
CG8710	Coilin	-	-	300	5	-	-	+	not expressed	
CG12165	INCENP	-	-	297	4	-	-	+	+	
CG12047	NuMA	-	-	285	5	-	-	-		
CG5595	Sex combs extra (Sce)	-	-	283	4	-	-	+	-	
CG12265	Deterin	-	-	238	3	-	-	+	-	
CG3836	Stonewall (Stwl)	-	-	237	5	-	-	+	+	
CG10632	Sosondowah (Sowah)	-	-	179	3	-	-	+	+	
CG5690	Centrobin	-	-	168	3	-	-	+	+	
CG3068	Aurora A	-	-	134	2	-	-	-		
CG8114	Pebble	-	-	106	1	-	-	+	not expressed	
CG17286	Spd2	-	-	88	2	-	-	-		
CG17033	Early girl	-	-	84	1	-	-	+	-	
CG34379	Shroom	-	-	70	1	-	-	+	not expressed	
CG6176	Grip75	-	-	40	1	62	1	+	-	+
CG7507	Dynein heavy chain 64C	-	-	-	-	299	6	-		
CG3210	Dynamin related protein 1	-	-	-	-	298	5	-		
CG9212	Nipsnap	-	-	-	-	286	5	+	-	+
CG1569	Rod	-	-	-	-	172	3	+		-
CG9375	Ras oncogene at 85D	-	-	-	-	155	2	+		not expressed
CG14999	Replication factor C su4 (Rfc4)	-	-	-	-	122	1	+	-	+
CG12244	Licorne (lic)	-	-	-	-	99	2	+	-	+
CG9900	Zeste-white 10 (Zw10)	-	-	-	-	90	1	+	-	+
CG7948	Spindle A	-	-	-	-	75	1	+	-	+
CG9193	PCNA	-	-	-	-	72	1	+		-
CG6875	asp	-	-	-	-	54	2	-		
CG4204	Elongin B	-	-	-	-	54	1	-		
CG18729	Zwilch	-	-	-	-	49	1	+	-	+
CG3157	gamma-Tub23C	-	-	-	-	40	2	+	-	+
CG7380	BAF	112	1	238	3	70	2	+	-	-

Table S1. A list of putative Falafel-interacting proteins identified by AP-MS.
Several cell cycle, DNA repair and development-related proteins were selected from the AP-MS experiments. As expected, PP4c and PP4R2 did not show interaction with EVH1 or Smk-1. CG number is the FlyBase accession number of the proteins; Score is Mascot scores, # pep is number of identified peptides. The in vitro protein-protein interaction section indicates which proteins were selected for in vitro binding experiments; + means (selected or interacting); - means (not selected or no interaction was detected); not expressed means that IVTT expression of the prey was not efficient or successful. Highlighted proteins form complexes: Orange are components of the Chromosomal Passenger Complex (CPC); Light blue are components of the Rod-Zwilch-Zw10 (RZZ) complex; Grey are components of the PP4 holoenzyme.

Table S2. List and IDs of *Drosophila* proteins used in this study

Name	CG number	Gold Collection ID	Uniprot ID
BAF	CG7380	GH06291	Q9VLU0
Borealin-related	CG4454	RE31802	Q9VLD6
CENP-C	CG31258	FI18815	Q9VHP9
Centrobin	CG5690	BS26913	Q9I7U5
CG8478	CG8478	LD23630	Q961C3
Coilin	CG8710	FI06021	A1Z7A8
Deterin	CG12265	RE55472	Q9VEM2
Early girl	CG17033	LD41235	Q9VUV7
Fascetto	CG11207	LD35624	Q9VZ62
Grip75	CG6176	FI23926	Q9VKU7
γTub23C	CG3157	LD40196	P23257
INCENP	CG12165	RE52507	A0A0B4LFQ2
Licorne	CG12244	SD04985	O62602
Miranda	CG12249	LD02989	Q8IN63
Nipsnap	CG9212	LD01807	Q9VXK0
Otefin	CG5581	LD41911	P20204
PCNA	CG9193	LD45889	P17917
Pebble	CG8114	MIP14634	D2NUE8
Prp16	CG32604	LD24737	Q9VY54
Psc	CG3886	MIP14548	P35820
Ras oncogene at 85D	CG9375	LD17536	P08646
RfC4	CG14999	LD40483	P53034
Sce	CG5595	LD23953	Q9VB08
Shroom	CG34379	GH26230	A1Z9P3
Sowah	CG10632	LD31582	Q960W9
Spindle A	CG7948	RE29170	Q27297
Staufen	CG5753	LP08764	P25159
Stonewall	CG3836	LD17962	Q9VUE5
Zeste-white 10	CG9900	FI17506	Q9W4X9
Zwilch	CG18729	RE71749	Q9VA00

Table S3. List of oligonucleotide primers used in this study.

Primer name	5'-3' sequence	Used for
Centrobin 36-39 AxPA fw	GATGCGTTGGCCGCCCCCCCGGCCCCTCCCCCGGC	In vitro mutagenesis
Centrobin 36-39 AxPA rev	GCCGGGGGAGGGGCCGGGGGGGCGGCCAACGCATC	
Centrobin 51-54 AxxA fw	CAGCGTACCAGGCCCTACATGCCAGCAAGAAATC	In vitro mutagenesis
Centrobin 51-54 AxxA rev	GATTTCTTGCTGGCATGTAGGGCCTGGTACGCTG	
CG8478 1-250 fw NotI	ATGCGCGGCCGCATGGACGATTCGCGCAGAAC	Cloning to pHY22
CG8478 1-250 rev BamHI	GCGCGGATCCCTACACAGAATCAGCGGAGATC	
CG8478 162-411 fw NotI	GCATGCGGCCGCATGACGGTTAAGGCCAATACCC	Cloning to pHY22
CG8478 162-411 rev BamHI	GCATGGATCCTCACGGATTGCTGTTCAGACGC	
CG8478 323-571 fw NotI	TATAGCGGCCGCATGTCACTAAGCCGTCGCAGCTG	Cloning to pHY22
CG8478 323-571 rev BamHI	GCGCGGATCCTTAATCGTGGGCACTCTGTG	
CG8478 504-507 AxPA fw	GAACCCAGTCCGCCGCTCCAGCCAGCGTGAAAAAAATTCC	In vitro mutagenesis
CG8478 504-507 AxPA rev	GGAATTTTTTTCACGCTGGCTGGAGCGGCGGACTGGGTTC	
Flfl 1-168 L70A fw	CAACAGGATACGGCCATCGTGTGGTCC	In vitro mutagenesis
Flfl 1-168 L70A rev	GGACCACACGATGGCCGTATCCTGTTG	
Grip75 1-100 IVTT mmg rev	TCATTACTATCACCCCATCATCAGACGTCCGATCTCAGCGT	PCR fragment for IVTT
Grip75 1-100 IVTT T7 fw	GGGAATTAATACGACTCACTATAGGGAGAGCCGCCACCATGATACACGATTTGTTATT	
Grip75 1-267 fw NotI	GCAGGCGGCCGCATGATACACGATTTGTTATTG	Cloning to pHY22
Grip75 1-267 rev BamHI	ATGCGGATCCTTAGTTGCGCCCCATTTTGAAG	
Grip75 181-280 IVTT mmg rev	TCATTACTATCACCCCATCATTTTTGCCGCCAGTGGGTCGG	PCR fragment for IVTT
Grip75 181-280 IVTT T7 fw	GGGAATTAATACGACTCACTATAGGGAGAGCCGCCACCATGGCTCACTGGCTGTTGTTTGG	
Grip75 183-449 fw NotI	ATATGCGGCCGCATGTGGCTGTTGTTTGGAGTGAT	Cloning to pHY22
Grip75 183-449 rev BamHI	ATGCGGATCCTTAGTACCGCTCGATCGTTGTTG	
Grip75 369-650 fw NotI	ATATGCGGCCGCATGGTCGGTACCATGGAAAC	Cloning to pHY22
Grip75 369-650 rev BamHI	GCGCGGATCCTTATCATGCGGACGTATTATGAC	
Grip75 91-190 IVTT mmg rev	TCATTACTATCACCCCATCATGTCAATCACTCCAAACAACA	PCR fragment for IVTT
Grip75 91-190 IVTT T7 fw	GGGAATTAATACGACTCACTATAGGGAGAGCCGCCACCATGGAGGAGTACTACGCTGAGAT	
Hs SMEK1 EVH1 L69A fw	CAACAGGACACTGCCATTGTGTGGTC	In vitro mutagenesis
Hs SMEK1 EVH1 L69A rev	GACCACACAATGGCAGTGTCCTGTTG	
Hs SMEK1 EVH1 GW fw	GGGGACAAGTTTGTACAAAAAAGCAGGCTTAATGACCGACACCCGGCGGCGGGTG	Gateway cloning
Hs SMEK1 EVH1 GW rev	GGGGACCACTTTGTACAAGAAAGCTGGGTATCAAGATTCATCCACAAGGTCCTGAGTG	
Incenp 120-123 AxPA fw	GGCCTCGCTGGCCCCGCCTGCCCCTGTGCCCG	In vitro mutagenesis
Incenp 120-123 AxPA rev	CGGGCACAGGGGCAGGCGGGGCCAGCGAGGCC	
Incenp 492-495 AxxA fw	CTGTTGGTCGCGCCTTCACAGCCACTCAAACGAC	In vitro mutagenesis
Incenp 492-495 AxxA rev	GTCGTTTGAGTGGCTGTGAAGGCGCGACCAACAG	
Incenp 653-656 AxPA fw	CAAGCCCAAGAAGGCCCTTCCGGCCCCGCCGAAAAC	In vitro mutagenesis
Incenp 653-656 AxPA rev	GTTTTCGGCGGGGCCGGAAGGGCCTTCTTGGGCTTG	
Incenp 1-329 fw NotI	ATATGCGGCCGCATGGAGGACATCTTGGGCG	Cloning to pHY22
Incenp 1-329 rev BamHI	GCGCGGATCCTCACTCCTGAACCGTTCGCGAAT	
Incenp 223- 534 fw NotI	GCAAGCGGCCGCATGGTGAACACCACGAAGACAC	Cloning to pHY22
Incenp 223- 534 rev BamHI	GCGCGGATCCTCAGACCGACCTTAGGTTCGTAC	
Incenp 445- IVTT T7 fw	GGGAATTAATACGACTCACTATAGGGAGAGCCGCCACCATGAAAATGCGCGTGGAGGCGTT	PCR fragment for IVTT
Incenp 445-755 fw NotI	GCTAGCGGCCGCATGAAAATGCGCGTGGAGGCGTT	Cloning to pHY22
Incenp 445-755 rev BamHI	GCGCGGATCCCTAGTATTTCGGCAGCTCGG	
Incenp 534-634 fw NotI	GCGCGCGGCCGCAAATGGTCAATTCCACGTCCACA	Cloning to pHY22
Incenp 534-634 rev BamHI	GCATGGATCCTTACTTGGCCTTCTTGAGCGCCT	
Incenp 534-634 IVTT mmg rev	TCATTACTATCACCCCATCATCTTGGCCTTCTTGAGCGCCT	PCR fragment for IVTT
Incenp 534-634 IVTT T7 fw	GGGAATTAATACGACTCACTATAGGGAGAGCCGCCACCATGGTCAATTCCACGTCCACAAA	
Incenp 600-699 fw NotI	GCGCGCGGCCGCATGGAGGAGCGAAAGCGAATTGA	Cloning to pHY22
Incenp 600-699 rev BamHI	GCGCGGATCCTTAAATTGCCTCTCCCCTTACGT	
Incenp 600-699 IVTT mmg rev	TCATTACTATCACCCCATCATAATTGCCTCTCCCCTTACGT	PCR fragment for IVTT
Incenp 600-699 IVTT T7 fw	GGGAATTAATACGACTCACTATAGGGAGAGCCGCCACCATGGAGGAGCGAAAGCGAATTGA	
Incenp 657-755 IVTT mmg rev	TCATTACTATCACCCCATCATCTAGTATTTCGGCAGCTCGG	PCR fragment for IVTT
Incenp 657-755 IVTT T7 fw	GGGAATTAATACGACTCACTATAGGGAGAGCCGCCACCATGCCGCCGAAAACCAAGTACAC	
Licorne 102-233 fw NotI	ATATGCGGCCGCATGTGCCCCTACACCGTCCAC	Cloning to pHY22
Licorne 102-233 rev BamHI	ATGCGGATCCTTAAACATCCGACCTGATGTCG	
Licorne 1-132 fw NotI	ATATGCGGCCGCATGTCCAAACGCCACCGCCTC	Cloning to pHY22
Licorne 1-132 rev BamHI	GCATGGATCCTTAGAACTTGTCCAGGCTGGTGC	
Licorne 204-334 fw NotI	ATATGCGGCCGCATGACCATTGATGCCGGTTGCAAG	Cloning to pHY22
Licorne 204-334 rev BamHI	ATATGGATCCCTACTGCGCCGGCTGCGCG	
Mira 77-80 AxxA fw	GGCCAAGGAAGCCGCCATTGCCAAGGAGGACAAAAAG	In vitro mutagenesis
Mira 77-80 AxxA rev	CTTTTTGTCCTCCTTGGCAATGGCGGCTTCCTTGGCC	
Mira 92-95 AxxA fw	GATTACGTCTGGCCCGTACTGCCTCGTTGCCGC	In vitro mutagenesis
Mira 92-95 AxxA rev	GCGGCAACGAGGCAGTACGGGCCAGACGTAATC	
Nipsnap 1-100 fw NotI	GCATGCGGCCGCATGCTTAAGCTACGTAATC	Cloning to pHY22
Nipsnap 1-100 rev BamHI	GCATGGATCCTTATGATGCCACCAATTCGCAG	
Nipsnap 172-273 fw NotI	ATATGCGGCCGCATGAACATTTACGAAATGCGCTC	Cloning to pHY22
Nipsnap 172-273 rev BamHI	ATGCGGATCCTTATTGTGAGGGCGAGAACTC	
Nipsnap 79-194 fw NotI	GCATGCGGCCGCATGTACAAAACTACTGTCGCTTTG	Cloning to pHY22
Nipsnap 79-194 rev BamHI	ATATGGATCCTTAGGCCCAGTTGTTTCCCCAC	
Prp16 113-251 fw NotI	ATATGCGGCCGCAAATGCTGCGCGAGCACAAAGAC	Cloning to pHY22
Prp16 113-251 rev BamHI	GCATGGATCCTTATGGCATATCCCAGTCCGACT	
Prp16 1-134 rev EcoRV	GCGCGATATCTTACATACGCTCGCGTGCCTTCTCGG	Cloning to pHY22
Prp16 1-468 fw EcoRV	GCGCGATATCATGTCCGACGACGACGAGTC	Cloning to pHY22
Prp16 1-468 rev EcoRV	GCGCGATATCTTACTCCCAGTGCTTCTTTTGCG	
Prp16 1-90 IVTT mmg rev	TCATTACTATCACCCCATCATAGAGGCGTTGGCCTGCGGCG	PCR fragment for IVTT
Prp16 1-90 IVTT T7 fw	GGGAATTAATACGACTCACTATAGGGAGAGCCGCCACCATGTCCGACGACGACGAGTC	
Prp16 224-357 fw EcoRV	ATATGATATCATGGGCAGCAGCGGTGGCATATC	Cloning to pHY22
Prp16 224-357 rev EcoRV	GCGCGATATCTTACATCATCTTCTGCTCCAGCTGCTCTT	
Prp16 357-845 fw EcoRV	GCGCGATATCATGAAGAGAACAAAGAGAATCAG	Cloning to pHY22
Prp16 357-845 rev EcoRV	GCGAGATATCTTAATAGATCTGCAGGGCGTCC	

Prp16 40-43 AxxA fw	GTGGCGGTGGAGCCAAGGTGGCCCAGGGATCGATG	In vitro mutagenesis
Prp16 40-43 AxxA rev	CATCGATCCCTGGGCCACCTTGGCTCCACCGCCAC	
Prp16 45-134 IVTT mmg rev	TCATTACTATCACCCCATCATACGCTCGCGTGCCTTCTCGG	PCR fragment for IVTT
Prp16 45-134 IVTT T7 fw	GGGAATTAATACGACTCACTATAGGGAGAGCCGCCACCATGGGATCGATGTTGGGCCTGGA	
Prp16 756-1222 fw EcoRV	GCGGGATATCATGGAGGTGACGTGCGAGGTGC	Cloning to pHY22
Prp16 756-1222 rev EcoRV	GCGCGATATCTCAAAGGCCAATCCTGGCC	
Prp16 98-101 AxxA fw	GTGAGTTTGCCGCCAAGAAAGCCGACACCAAGAG	In vitro mutagenesis
Prp16 98-101 AxxA rev	CTCTTGGTGTCGGCTTTCTTGGCGGCAAACTCAC	
Psc 1009-1601 fw NdeI	GCGCCATATGATGCCCACCTATAAGTATACACC	Cloning to pHY22
Psc 1009-1601 rev NcoI	GCGCCCATGGTCACTTGCTTTTGGTG	
Psc 1048-1051 AxxA fw	CTTGGCGGATTAGCCCCCTCAGCCCCCACCAAGTC	In vitro mutagenesis
Psc 1048-1051 AxxA rev	GACTTGGTGGGGGCTGAGGGGGCTAATCCGCCAAG	
Psc 1-594 fw NdeI	GCGCCATATGATGATGACGCCAGAATCGAA	Cloning to pHY22
Psc 1-594 rev NcoI	GCGCCCATGGTTACAGCTTGATATTGGGCTCCG	
Psc 505-1098 fw NdeI	GCGCCATATGATGTCGAAGGTGGAACCTGTGTC	Cloning to pHY22
Psc 505-1098 rev NcoI	GCAGCCATGGTTACACCTGCTGCTTTTCGGGCG	
Psc 594-750 fw NdeI	GCGCCATATGATGCTGAAAATCGATCTGTCCAA	Cloning to pHY22
Psc 594-750 rev NcoI	GCGCCCATGGTTATTCATAAGAATACTGTCGTC	
Psc 721-810 IVTT mmg rev	TCATTACTATCACCCCATCATTTTTTCTATCTTTGGAGATG	PCR fragment for IVTT
Psc 721-810 IVTT T7 fw	GGGAATTAATACGACTCACTATAGGGAGAGCCGCCACCATGAGAGCCATCACCCCGCCTTC	
Psc 721-876 fw NdeI	GCGCCATATGATGAGAGCCATCACCCCGCCTTC	Cloning to pHY22
Psc 721-876 rev NcoI	GCGCCCATGGTTAGGCAAAGGGATTTGGTC	
Psc 749-752 AxPA fw	CAGTATTCTTGCCAAACCGGCCAGCTGCATGC	In vitro mutagenesis
Psc 749-752 AxPA rev	GCATGCAGCTGGCCGGTTTGGCAAGAATACTG	
Psc 786-875 IVTT mmg rev	TCATTACTATCACCCCATCATGGCAAAGGGATTTGGTCGCG	PCR fragment for IVTT
Psc 786-875 IVTT T7 fw	GGGAATTAATACGACTCACTATAGGGAGAGCCGCCACCATGACGGTGGACTTTAAGATACG	
Psc 813-816 AxPA fw	GAAAAACCGCTGGCCCCACCGGCCGCAAAGCCGC	In vitro mutagenesis
Psc 813-816 AxPA rev	GCGGCTTTGCGGCCGGTGGGGCCAGCGGTTTTTC	
Psc 833-836 AxxA fw	CTCCGCTCAGGCCGCGCCGGCCCCATCGCCGATAC	In vitro mutagenesis
Psc 833-836 AxxA rev	GTATCGGCGATGGGGCCGGCGCGGCCTGAGCGGAG	
Psc 847-1009 fw NdeI	GCGCCATATGATGCAGATGTCAGCTCCTGGTAA	Cloning to pHY22
Psc 847-1009 rev NcoI	GCGCCCATGGTTAGGGCATTGTTGGAATATTGT	
RfC4 101-226 fw NotI	ATGAGCGGCCGCATGAAGATGTTTGCTCAGCAGAAG	Cloning to pHY22
RfC4 101-226 rev BamHI	ATGCGGATCCTTAGATGTCGCCAAATCCCTGAG	
RfC4 1-126 fw NotI	ATATGCGGCCGCATGCCGGAAGAACCAGAGAA	Cloning to pHY22
RfC4 1-126 rev BamHI	ATATGGATCCTTACATGCTGTCCGCCTCGTCC	
RfC4 206-331 fw NotI	ATATGCGGCCGCATGCAAGGTGATATGCGACAGGG	Cloning to pHY22
RfC4 206-331 rev BamHI	ATATGGATCCCTAATGCTTCTCGGCGGCAA	
Sowah 107-110 AxxA fw	CGCATCTCTTAACGCCGACTCGGCCATGAGGCAGCC	In vitro mutagenesis
Sowah 107-110 AxxA rev	GGCTGCCTCATGGCCGAGTCGGCGTTAAGAGATGCG	
Sowah 1-276 fw NotI	GCTAGCGGCCGCATGGAGCTACCGAAGGAGTT	Cloning to pHY22
Sowah 1-276 rev BamHI	GCGAGGATCCTTACAGCGTAACCTCCGGTGAATC	
Sowah 183-474 fw NotI	AGCGGCGGCCGCATGTCCATCGATGTGATCGACAAC	Cloning to pHY22
Sowah 183-474 rev BamHI	GCAGGGATCCTTACTGATGAGGGGCTCGGTGAG	
Sowah 357-635 fw NotI	GCAGGCGGCCGCATGACCCAGTTTGGTAGGGATAA	Cloning to pHY22
Sowah 357-635 rev BamHI	GCGCGGATCCTTAGGATTGCGAAGGATTGC	
Sowah 474-574 fw NotI	GCGCGCGGCCGCATGCAGCGTCATCATCACCACGT	Cloning to pHY22
Sowah 474-574 rev BamHI	GCGCGGATCCTTACGAGGAGCAGATGGAATCAC	
Sowah 474-574 IVTT mmg rev	TCATTACTATCACCCCATCATCGAGGAGCAGATGGAATCAC	PCR fragment for IVTT
Sowah 474-574 IVTT T7 fw	GGGAATTAATACGACTCACTATAGGGAGAGCCGCCACCATGCAGCGTCATCATCACCACGT	
Sowah 537-635 IVTT mmg rev	TCATTACTATCACCCCATCATTTAGGATTGCGAAGGATTGC	PCR fragment for IVTT
Sowah 537-635 IVTT T7 fw	GGGAATTAATACGACTCACTATAGGGAGAGCCGCCACCATGCTAATGCCGCCGCCCAAGGC	
Sowah 538-541 AxPA fw	GGAGGACCTAGCCCCGCCGGCCAAGGCGGTGG	In vitro mutagenesis
Sowah 538-541 AxPA rev	CCACCGCCTTGGCCGGCGGGGCTAGGTCCTCC	
Spindle-A 109-228 IVTT mmg rev	TCATTACTATCACCCCATCATATCGGATCTGTAGAGCGCCA	PCR fragment for IVTT
Spindle-A 109-228 IVTT T7 fw	GGGAATTAATACGACTCACTATAGGGAGAGCCGCCACCATGCTGCTGGGCGGCGGCATTGA	
Spindle-A 1-120 IVTT mmg rev	TCATTACTATCACCCCATCATGGTAATGGATCCCGTCTCAA	PCR fragment for IVTT
Spindle-A 1-120 IVTT T7 fw	GGGAATTAATACGACTCACTATAGGGAGAGCCGCCACCATGATGGAGAAGCTAACGAATGT	
Spindle-A 217-336 IVTT mmg rev	TCATTACTATCACCCCATCATGCTCTCCCTGGCGTCTCCTA	PCR fragment for IVTT
Spindle-A 217-336 IVTT T7 fw	GGGAATTAATACGACTCACTATAGGGAGAGCCGCCACCATGATTGTGGACAGTGCCATGGC	
Stwl 407-410 AxxA fw	GGATGACATCGCCCCGAGAGCCGCGGTGCCATC	In vitro mutagenesis
Stwl 407-410 AxxA rev	GATGGCACCGCGGCTCTCGGGGCGATGTCATCC	
Stwl 750-1037 EcoRV rev	GCGCGATATCTTAAACACCGAGTATGTCAT	Cloning to pHY22
Stwl 750-1037 NotI fw	TAGCGCGGCCGCATGTTAGCGGCTATGGGTGGTAA	
Stwl 882-885 AxPA fw	CTGGGCATGGAGCCGTGCCAGCCACCCAACGCC	In vitro mutagenesis
Stwl 882-885 AxPA rev	GGCGTTGGGTGGCTGGCACGGCTCCATGCCCAG	
Stwl 950-953 AxxA fw	CTTCAATGACGCCGCCCAGGCCAAGCCCGCTG	In vitro mutagenesis
Stwl 950-953 AxxA rev	CAGCGGGCTTGGCCTGGGCGGCGTCATTGAAG	
Zw10 1-187 fw NotI	ATATGCGGCCGCATGGAGGAAGAGGCGCCGCG	Cloning to pHY22
Zw10 1-187 rev BamHI	GCGCGGATCCTTAGTATTTCACCTTGATGGTC	
Zw10 144-337 fw NotI	ATATGCGGCCGCATGGACCTTGTCGGTCGTTTGAG	Cloning to pHY22
Zw10 144-337 rev BamHI	GCGCGGATCCTTACATTTTATCCTTCACATGATCGC	
Zw10 238-241 AxxA fw	GTGCAGGCTCTCGCCCAGGAGGCCTACAATCCGGC	In vitro mutagenesis
Zw10 238-241 AxxA rev	GCCGGATTGTAGGCCTCCTGGGCGAGAGCCTGCAC	
Zw10 294-485 fw NotI	ATATGCGGCCGCATGCTTCGCCCGAACTACAAGC	Cloning to pHY22
Zw10 294-485 rev BamHI	ATATGGATCCTTATCCGTGGGCTGGCGAAGAA	
Zw10 438-441 AxxA fw	CAAACGATCCCGCCCTCTTCGCCCGCTGCATGATC	In vitro mutagenesis
Zw10 438-441 AxxA rev	GATCATGCAGCGGGCGAAGAGGGCGGGATCGTTTG	
Zw10 GW fw	GGGGACAAGTTTGTACAAAAAAGCAGGCTTAATGGAGGAAGAGGCGCCGCGGTTC	Gateway cloning
Zw10 GW rev	GGGGACCACTTTGTACAAGAAAGCTGGGTATTATCCGTGGGCTGGCGAAGAATG	
Zwilch 1-220 IVTT mmg rev	TCATTACTATCACCCCATCATAGCACTAGAGGTCCAGGAGC	PCR fragment for IVTT
Zwilch 1-220 IVTT T7 fw	GGGAATTAATACGACTCACTATAGGGAGAGCCGCCACCATGTCGGCTTCTGCGAACTT	
Zwilch 218-431 IVTT mmg rev	TCATTACTATCACCCCATCATCAACAGATTGGTGCTGCACA	PCR fragment for IVTT
Zwilch 218-431 IVTT T7 fw	GGGAATTAATACGACTCACTATAGGGAGAGCCGCCACCATGCTACGCTGCTCCTGGACCTC	

Zwilch 422-641 IVTT rev	TCATTACTATCACTTAAGTGAGCGAATCTTGT	PCR fragment for IVTT
Zwilch 422-641 IVTT T7 fw	GGGAATTAATACGACTCACTATAGGGAGAGCCGCCACCATGGAGAGCAAGATGTGCAGCAC	
Zwilch GW fw	GGGGACAAGTTTGTACAAAAAAGCAGGCTTAATGTCGGCTTCTGCGAACTTGGCA	Gateway cloning
Zwilch GW rev	GGGGACCACTTTGTACAAGAAAGCTGGGTATTACTTAAGTGAGCGAATCTTGTG	
